# DHX15 and Rig-I Coordinate Apoptosis and Innate Immune Signaling by Antiviral RNase L

**DOI:** 10.3390/v16121913

**Published:** 2024-12-13

**Authors:** Barkha Ramnani, Trupti Devale, Praveen Manivannan, Aiswarya Haridas, Krishnamurthy Malathi

**Affiliations:** 1 Department of Biological Sciences, University of Toledo, 2801 West Bancroft Street, Toledo, OH 43606, USA; barkhar@umich.edu (B.R.); trupti.devale@rockets.utoledo.edu (T.D.); manivann@umich.edu (P.M.); aiswarya.pazhoormattomharidas@rockets.utoledo.edu (A.H.); 2 Department of Internal Medicine, University of Michigan, Ann Arbor, MI 48109, USA; 3 Department of Microbiology and Immunology, University of Michigan Medical School, Ann Arbor, MI 48109, USA

**Keywords:** RNase L, apoptosis, Rig-I, DHX15, innate immune signaling

## Abstract

During virus infection, the activation of the antiviral endoribonuclease, ribonuclease L (RNase L), by a unique ligand 2′-5′-oilgoadenylate (2-5A) causes the cleavage of single-stranded viral and cellular RNA targets, restricting protein synthesis, activating stress response pathways, and promoting cell death to establish broad antiviral effects. The immunostimulatory dsRNA cleavage products of RNase L activity (RL RNAs) recruit diverse dsRNA sensors to activate signaling pathways to amplify interferon (IFN) production and activate inflammasome, but the sensors that promote cell death are not known. In this study, we found that DEAH-box polypeptide 15 (DHX15) and retinoic acid-inducible gene I (Rig-I) are essential for apoptosis induced by RL RNAs and require mitochondrial antiviral signaling (MAVS), c-Jun amino terminal kinase (JNK), and p38 mitogen-activated protein kinase (p38 MAPK) for caspase-3-mediated intrinsic apoptosis. In RNase L-activated cells, DHX15 interacts with Rig-I and MAVS, and cells lacking MAVS expression were resistant to apoptosis. RL RNAs induced the transcription of genes for IFN and proinflammatory cytokines by interferon regulatory factor 3 (IRF-3) and nuclear factor kB (NF-kB), while cells lacking both DHX15 and Rig-I showed a reduced induction of cytokines. However, apoptotic cell death is independent of both IRF-3 and NF-kB, suggesting that cytokine and cell death induction by RL RNAs are uncoupled. The RNA binding of both DHX15 and Rig-I is required for apoptosis induction, and the expression of both single proteins in cells lacking both DHX15 and Rig-I is insufficient to promote cell death by RL RNAs. Cell death induced by RL RNAs suppressed Coxsackievirus B3 (CVB3) replication, and inhibiting caspase-3 activity or cells lacking IRF-3 showed that the induction of apoptosis directly resulted in the CVB3 antiviral effect, and the effects were independent of the role of IRF-3.

## 1. Introduction

Higher eukaryotes have evolved self-defense strategies for protection against viruses. Innate immune responses are essential for the initial detection of viruses and subsequent activation of adaptive immunity. Host antiviral responses are initiated through the recognition of viral pathogen-associated molecular patterns (PAMPs) by pattern-recognition receptors (PPRs) distributed in a cell-type-specific manner and in specific cellular compartments [1,2,3]. The replication of RNA viruses results in the synthesis of dsRNA replication intermediates that can be detected by PAMP receptors, like TLR3, in the endosome and RNA helicases, like Rig-I or MDA5, in the cytosol [4,5,6]. Rig-I and MDA5 contain a C-terminal DExD/H RNA helicase domain that binds the RNA ligand and an N-terminal caspase recruitment domain (CARD) that mediates signaling by interaction with the CARD domain of the mitochondrial adaptor protein, IFN-β promoter stimulator-1 (IPS-1/MAVS/VISA/Cardif), which is essential for triggering a signaling cascade that activates NF-κb and IRF-3 to induce the production of type I interferons (IFN), pro-inflammatory cytokines, and mitogen-activated protein kinase (MAPK) pathways [7,8,9,10,11,12,13,14,15,16,17,18,19]. Type I IFN activates intracellular signaling pathways via the engagement of the type I IFN receptor (IFNAR) complex, leading to the upregulation of a set of interferon-stimulated genes (ISGs), which are essential for antiviral activities [20]. In addition, dsRNA is sensed by diverse RNA binding proteins and RNA helicases, resulting in the activation of stress response pathways and apoptosis, causing the premature elimination of virus-infected cells, limiting viral replication and promoting antiviral defense [21].

Several members of the DExD/H box RNA helicases can sense cytosolic viral RNA and play important roles in antiviral signaling. Rig-I-like receptors (RLR) Rig-I and melanoma differentiation-associated protein 5 (MDA5) are members of cytoplasmic DExD/H RNA helicases that bind distinct types of RNAs based on preferences for the 5′ end structure, the length of dsRNA, and the secondary structure, allowing for the differential recognition of RNA viruses [8,21]. RLR signaling is initiated by PAMP binding, the induction of ATPase activity, and changes in conformation, facilitating interaction with MAVS, which in turn, recruits TNF receptor-associated factor (TRAF) family proteins to activate TANK binding kinase 1 (TBK1) and transcription factors IRF-3 and NF-kB to induce innate immune genes, including type I IFN [2,4,7,20,22]. In addition to its primary role in cytokine induction, both Rig-I and MDA5 can induce apoptosis in response to viral RNA and RNA agonists in normal and tumor cells, and these effects are independent of IFN production [23,24,25]. The mechanisms underlying RLR-mediated apoptosis are diverse and include the transcriptional induction of proapoptotic genes, like the p53-upregulated modulator of apoptosis (PUMA), phorbol-12-myristate-13-acetate-induced protein 1 (PMAIP1, NOXA), the activation of tumor necrosis factor (TNF)-related apoptosis-inducing ligand (TRAIL) signaling pathways, the downregulation of anti-apoptotic genes, like B-cell leukemia/lymphoma 2 protein(bcl2), baculoviral IAP repeat-containing protein 3 (BIRC3), and IRF-3-mediated mitochondrial apoptosis by activating caspase-3 [23,24,26,27,28,29]. Besides Rig-I and MDA5, several non-RLR DExD/H helicases, like DDX1, DDX3, DHX9, DHX15, DDX21, DHX33, DHX36, DDX41, and DDX60, are involved in regulating antiviral signaling pathways. DDX3 interacts with MAVS, TRAF3, and TBK1 to induce interferon β production upon infection with Sendai Virus and *Listeria monocytogenes* and localizes to stress granules to regulate inflammasomes [30,31,32]. DDX1, DDX21, and DHX36 form a complex with TIR-domain-containing adapter-inducing interferon-β (TRIF) for binding dsRNA in dendritic cells [33]. DHX33 binds dsRNA and interacts with MAVS to activate the NOD-, LRR-, and Pyrin domain-containing protein 3 (NLRP3) inflammasome pathways [34,35]. DHX9 and DHX36 participate in TLR signaling by CpG DNA via myeloid differentiation primary response 88 (MYD88) [36,37]. DHX60 is an IFN-inducible RNA helicase that can sense dsRNA to promote RLR-dependent signaling [38].

DHX15 is a ubiquitously expressed protein found in many cellular compartments, where it functions in pre-mRNA splicing, ribosome biogenesis, tumorigenesis, and innate immune responses. In HeLa cells, DHX15 was found to interact with MAVS to activate NF-κB and MAPK signaling and not IRF-3 to induce proinflammatory cytokine expression and caspase-3-mediated apoptosis, respectively [39]. In myeloid dendritic cells, DHX15 interacts with DHX9 and is essential for the activation of IRF-3, NF-κB, and MAPK pathways in response to poly I:C and RNA virus infection [40]. In these cells, DHX15 specifically interacts with short poly I:C and is an essential sensor for detecting RNA virus infection. In intestinal epithelial cells, DHX15 interacts with NOD-, LRR-, and Pyrin domain-containing protein 6 (NLRP6) to transduce a signal through MAVS to upregulate type I and III interferons and trigger inflammasome activation [41]. More recently, DHX15 was identified as a coreceptor for RIG-I-mediated interferon signaling in response to RNA virus infection in HEK293 cells [42]. In other studies, DHX15 colocalized with NLRP6 and induced liquid–liquid phase separation (LLPS) during RNA virus infection to mount an effective immune response by serving as a signaling hub [43]. DHX15 contributes to carcinogenesis in breast, prostate, and leukemia and functions as a tumor suppressor in glioma and gastric cancer [44,45,46,47,48]. In contrast, DHX15 appears to have distinct roles in innate immunity as an RNA sensor that is driven by interaction with dsRNA ligands and promoting signaling events through protein–protein interactions [42].

The 2′, 5′-oligoadenylate synthetase (OAS)/RNase L system is an IFN-induced innate immune pathway that responds to dsRNA PAMPs to induce the degradation of viral and cellular RNAs, thereby blocking viral infection [49,50]. OAS enzymes are transcriptionally induced by IFN and activated by dsRNA to produce a unique 2′-5′-oligoadenylate, 2-5A [p*x*5 = A(2 = p5 = A)*n*; *x*_1 to 3; *n*_2], produced from cellular ATP [51]. The 2-5A binds to a latent endoribonuclease, RNase L, causing its dimerization and activation [52,53,54]. Active RNase L cleaves single-stranded viral and cellular RNAs, including rRNAs in intact ribosomes and mRNA on the 3′ end of UpAp and UpUp residues, resulting in cleaved RNAs that have double-stranded regions with a 5′-hydroxyl and a 2′3′-cyclic phosphoryl group [55]. Recent studies show that RNase L induces a ribotoxic stress response, likely through ribosome collisions, activating zipper sterile α-motif kinases (ZAK α), JNK, and p38 kinases [56,57]. In addition to the expected RNA decay, RNase L unexpectedly induces and alters the transcription of antiviral and proinflammatory genes and induces apoptosis [56,58,59,60]. The elimination of viral genomes and mRNA have a direct antiviral effect, and the byproducts of RNase L nucleolytic activity, RL RNAs, participate in signaling events engaging diverse dsRNA receptors [49,50]. The RL RNAs signal through Rig-I/MDA5 and MAVS to induce IFN production by promoting antiviral stress granule assembly through PKR activity [61,62], activate inflammasome through DHX33 and MAVS [35], induce autophagy, and promote the switch to apoptosis, involving JNK and PKR activity [59,63]. RNase L activity, therefore, causes direct and indirect antiviral effects by activating diverse dsRNA signaling pathways, but the receptors involved in apoptotic cell death are not known. Here, we show that RL RNAs induce intrinsic apoptosis through the coordinate activity of RNA helicases DHX15 and Rig-I and downstream MAVS, JNK, and p38 activation that is independent of IRF-3/NF-kB signaling and IFN and cytokine production. RNase L restricts CVB3 replication, and premature cell death induced by RL RNAs promotes antiviral effect. Antiviral role of RNase L in virus-infected cells is the result of coordination of multiple dsRNA signaling pathways to activate innate immune and cell death pathways to clear viruses.

## 2. Materials and Methods

### 2.1. Chemicals, Reagents, and Antibodies

Chemicals, unless otherwise indicated, were from Sigma-Aldrich (St. Louis, MO, USA). Antibodies against DHX15 (SC-271686), OAS1 (SC-98424), OAS2 (SC-374238), Total-p38 (SC-7972), IκBα (SC-371), GAPDH (SC-47724), and p65 (SC-109) were from Santa Cruz Biotechnology (Santa Cruz, Dallas, TX, USA). The Myc-tag sepharose beads used for immunoprecipitation were from Cell Signaling (Danvers, MA, USA). LC3 (2775), poly (ADP-ribose) polymerase (PARP) (9542), cleaved PARP (5625), caspase-3 (9662), cleaved caspase-3 (9664), FLAG (1479), Myc (2272), Phospho-JNK (9251), Total-JNK (9252), Phospho-p38 (9211), PKR (12297), RIG-I (3743), P-cJun (9261), P-MAPKAPK-2 (3007), IRF-3 (4302), Lamin A/C (2032), and β-actin (3700) were from Cell Signaling Technology (Danvers, MA, USA). Antibody against OAS3 (PA5-31090) was from Thermo Fisher Scientific (Waltham, MA, USA), and MAVS (ALX-210-929-C100) was purchased from Enzo Life Sciences (Farmingdale, NY, USA). Antimouse IgG and antirabbit IgG horseradish peroxidase (HRP)-linked secondary antibodies were from Cell Signaling Technology, and enhanced chemiluminescence (ECL) reagents were from Boston Bioproducts (Ashland, MA, USA) and Biorad (Hercules, CA, USA). IFN-β was from Biogen Idec (Cambridge, MA, USA). Puromycin (Millipore Sigma, St. Louis, MO, USA), JNK inhibitor 8 (Millipore Sigma, St. Louis, MO, USA), p38 inhibitor (Calbiochem, San Diego, CA, USA), zVAD-FMK (Calbiochem, San Diego, CA, USA), Ac-DEVD-CHO (Enzo Life Sciences), and necrostatin-1 (Santa Cruz Biotechnology, Dallas, TX, USA) were prepared and used at indicated concentrations. Poly I:C was purchased from Calbiochem (San Diego, CA, USA).

### 2.2. Cell Culture and Transfections

The human fibrosarcoma cell line, HT1080 (a gift from Ganes Sen, Cleveland Clinic, Cleveland, OH, USA), PKR KO [62], Rig-I KO [62], OAS1 KO, OAS2 KO, OAS3 KO (this study), IRF-3 KO, NuFF cells (a gift from Saurabh Chattopadhyay, University of Kentucky), and HeLa-M and HeLa-M cells expressing RNase L (a gift from Robert Silverman, Cleveland clinic) were cultured in Dulbecco’s modified minimal essential medium with 10% fetal bovine serum, 100 µg/mL penicillin/streptomycin, 2 mM L-glutamine, and nonessential amino acids. Cells were maintained in 95% air, 5% CO_2_ at 37 °C. Transfection of 2-5A (10 µM) was performed using lipofectamine 2000 (Invitrogen, Carlsbad, CA, USA), according to the manufacturer’s protocol. RNase L-cleaved small RNAs (RL RNAs) and control small RNAs (Ctrl RNAs) (2 µg/mL) were transfected using PolyJet reagent (SignaGen Laboratories, Frederick, MD, USA), according to the manufacturer’s protocol. In experiments involving inhibitors, cells were preincubated with inhibitors at indicated concentrations for 1 h prior to transfection and then replaced with growth medium.

### 2.3. 2-5A, RNase L-Cleaved Small RNAs and Control Small RNAs

2–5A (p_3_(A2′p)*_n_*A, where *n* = 1 to >3), was prepared enzymatically from ATP and recombinant 2–5A synthetase (a generous gift from Rune Hartmann, University of Aarhus, Aarhus, Denmark) as described previously [64]. RNase L-cleaved small RNAs (RL RNAs) and control small RNAs (Ctrl RNAs) were prepared as previously described. [61]. Briefly, total cellular RNA was isolated from HT1080 cells after transfection with 10 µM 2-5A (the source of RNase L-cleaved small RNAs) or mock-transfected cells (control small RNAs) using TRIzol reagent after 6 h. Activation of RNase L was determined by rRNA cleavage monitored on RNA chip using Bioanalyzer. Small-RNA cleavage products (<200 nucleotides) were purified using a solid-phase fractionation method (mirVana miRNA isolation kit; Ambion, Life Technologies, Carlsbad, CA, USA) as described previously [61]. The purified small RNAs were applied to cells by complexing with PolyJet reagent (SignaGen Laboratories, Frederick, MD, USA), according to the manufacturer’s protocol.

### 2.4. Generation of Gene Knockout Cell Lines Using CRISPR/Cas9 System

The *PKR* or *Rig-I* knockout HT1080 cells were constructed using the CRISPR/Cas9 gene editing system, as we described previously [62]. *OAS1*, *OAS2*, or *OAS3* knockout HT1080 cells were generated using small guide RNAs (sgRNAs) (Table 1) cloned into the vector pSpCas9(BB)-2A-Puro (PX459; Addgene plasmid 62988) V2.0 (a gift from Feng Zhang), which was prepared by digestion with BsmBI [65,66]. HT1080 cells were transfected with 2 μg of the resulting plasmids using PolyJet reagent and selected in 1 μg/mL puromycin. Single-cell clones were obtained by plating 2.5 cells/mL in a 48-well plate, and gene knockout clones were validated by immunoblotting and genomic sequencing.

### 2.5. Plasmid Constructs and Site-Directed Mutagenesis

Human DHX15 cDNA was cloned from HT1080 cells by reverse transcription and cDNA synthesis and subcloned into vector pKMyc (a gift from Ian Macara (Addgene plasmid #19400; http://n2t.net/addgene, accessed on 19 November 2020 RRID:Addgene_19400) at Nhe I and Not I restriction sites using forward primer 5′ GCGTAGCTAGCATGTCCAAGCGGCAC 3′ and reverse primer 5′ AAGCGGCCGCTCAGTACTGTGAATATTCCT 3′. Site-directed mutagenesis of human DHX15 was performed using pKMyc-DHX15 construct and the QuikChange Lightning site-directed mutagenesis kit, according to the manufacturer’s instructions (Agilent Technologies, Santa Clara, CA, USA) using the specific primer sequences (Table 2). All constructs were verified by nucleotide sequencing, and expression was confirmed by Western blot analysis. Plasmids FLAG-Rig-I, FLAG Rig-I KK858/861AA (Takashi Fujita, University of Tokyo, Tokyo, Japan), FLAG-IkB-SR (Brian Ashburner, University of Toledo, OH, USA), IFN-β-luc (Michael Gale, University of Washington, WA, USA), IP10-luc, NF-kB-luc, IL-8-luc (George Stark, Cleveland Clinic, OH, USA), and CCL5-luc (Katherine Fitzgerald, University of Massachusetts, Worcester, MA, USA) were transfected using PolyJet reagent, as per the manufacturer’s instructions.

### 2.6. Lentivirus Transduction and siRNA Gene Silencing

pLKO.1 plasmid with short hairpin RNA targeting DHX15 (DHX15 LV1 (TRCN0000000006), DHX15 LV2 (TRCN0000000007), DHX15 LV3 (TRCN0000000010), DHX15 LV4 (TRCN0000425479), DHX15 LV5 (TRCN0000420388), and MAVS (TRCN0000148945) was purchased from Millipore Sigma (St. Louis, MO, USA). For pseudo lentivirus packaging in HEK293T cells, the shRNA lentivirus plasmid (2 μg) and packaging plasmid (psPAX2 (2 μg) and pMD2.G (1 μg) obtained from Addgene) were co-transfected using lipofectamine 2000 reagent. Lentivirus particles were harvested at 48–96 h post-transfection. The supernatant containing lentivirus was collected, centrifuged to remove cell debris, and stored at −80 °C until further use. To generate stable cell lines, HT1080 cells in 12-well plates were transduced with 100 μL of pseudo lentivirus in the presence of polybrene (5 μg/mL). After 48 h, cells were selected in 1 μg/mL puromycin in complete medium. Single-cell clones were obtained by plating 2.5 cells/mL in a 48-well plate, gene knockout clones were validated by immunoblotting, and multiple clones were analyzed. Transient gene knockdown in indicated cells was achieved using gene-specific siRNA targeting Rig-I (L-012511-00-0005, Horizon Discovery, Lafayette, CO, USA), Mda5 (L-013041-00-0005, Horizon Discovery, Lafayette, CO, USA), DHX15 (4392422, Thermofisher Scientific, Life Technologies, Carlsbad, CA, USA), or IRF-3 (Cell Signaling Technology, Danvers, MA, USA, transfected using Lipofectamine 2000 reagent at a final concentration of 5 nM to 50 nM, according to the manufacturer’s instructions. Knockdown efficiency was determined by comparing protein levels to control cells on immunoblots using specific antibodies.

### 2.7. Coxsackievirus B3 (CVB3) Growth and Titration

HeLa cells were used to generate CVB3 viral stocks and for plaque assays. CVB3 stocks were produced by the transfection of HeLa cells with linearized plasmid pH3 (obtained from Lindsay Whitton, The Scripps Research Institute, CA), which encodes an infectious clone of CVB3. After 24–30 h, when 50% of cells showed cytopathicity and rounded up, cells were collected and freeze–thawed three times on dry ice to release the virions. HeLa cells in T75 flasks that were about 80% confluent were infected with the freeze–thawed supernatants, and the virus was harvested after 24 h by freeze–thaw cycles, clarified to remove cell debris, aliquoted, and frozen. Recombinant CVB3-GFP (obtained from Lindsay Whitton, The Scripps Research Institute, CA) stock was generated as wtCVB3 with the only change in cells harvested at 48 h. Viral titers were determined by plating HeLa cells (3 × 10^5^/well) in 6-well plates and infected the next day with a 10-fold serial dilution of virus in serum-free medium. After 1 h of adsorption, the cells were overlaid with DMEM containing 0.5% carboxymethylcellulose and incubated for 48 h. The cells were fixed with 10% formaldehyde in PBS and stained using 0.1% crystal violet in 20% ethanol and washed in water, and the plaques were counted. The assays were performed in triplicate, and the fold change in virus titers was determined from three independent experiments. CVB3 genomic RNA copy numbers were determined by quantitative real-time reverse transcriptase PCR using CVB3-specific primers as described [67]. RNA from the supernatants of infected cells was isolated using Trizol LS reagent (Invitrogen) or the QIAmp viral RNA kit (Qiagen, Valencia, CA, USA) for cDNA synthesis using random decamers and the RETROscript cDNA synthesis kit (Invitrogen) and PCR using SYBR green reagents (Biorad, Hercules, CA, USA) using CVB3 forward (5′-CACACTCCGATCAACAGTCA 3′) and CVB3 reverse primer (5′-GAACGCTTTCTCCTTCAACC 3′). Copy numbers were interpolated from an in vitro transcribed pH3 CVB3 RNA standard and expressed as copy number per ml of supernatant. All assays were performed in triplicate, and the fold change in CVB3 RNA copies was determined from three independent experiments. In some experiments, GFP+ plaques were counted using 4× magnification and the EVOS M5000 Imaging system (Thermoscientific, Life Technologies, Carlsbad, CA, USA) and compared to numbers in the plaque assay.

### 2.8. Cell Death Assays

Cell viability was determined using the colorimetric Cell Titer 96 Aqueous Cell Proliferation Assay (Promega, Madison, WI, USA). Briefly, cells (8 × 10^3^) were seeded into 96-well plates and transfected with 2-5A (10 µM) with Lipofectamine 2000 or RL RNAs and Ctrl RNAs (2 µg/mL) using PolyJet reagent. In some experiments, indicated plasmids were transfected prior to RL RNA or Ctrl RNA. At indicated times, 20 µL of tetrazolium salt (MTS reagent) was added and incubated at 37 °C. Absorbance was measured at 490 nm with a 96-well plate reader (model Spectra Max iD5; Molecular Devices, Menlo Park, CA, USA). Cell viability was normalized against mock-treated cells. For the trypan blue exclusion experiments, cells treated as above were stained in 0.4% trypan blue solution, and viable cells were determined using Countess 3 Automated Cell Counters (Life Technologies, Carlsbad, CA, USA) and normalized to mock-treated cells.

### 2.9. Real-Time Monitoring of Cell Death Using Dual Dyes

Cell death was analyzed in real time using dual dyes and an Incucyte S3 Live-Cell imaging system (Essen Bio Science, Ann Arbor, MI, USA). Cells (8 × 10^3^) were seeded into a 96-well plate and transfected with RL RNAs and Ctrl RNAs (2 µg/mL) using PolyJet reagent. Cells were incubated with 250 nM Sytox-Green cell-impermeable nucleic acid dye (Thermo Fisher Scientific, Waltham, MA, USA) that indicates dead cells and 250 nM of SytoTM 60-Red cell-permeable dye (ThermoFisher scientific, Waltham, MA, USA), which quantifies the total number of cells present in each field, and images were obtained in real time as indicated. Cell death was quantified as the percent of Sytox-Green-positive dead cells normalized to the total number of cells that were stained Sytox-Red-positive at each time point using Incucyte S3 software 2021C. The results shown are representative of four values per well performed in triplicate from three experimental samples and shown as mean ± SD.

### 2.10. Caspase-3/7 Assay

Cells (8 × 10^3^) were grown in black-walled 96-well plates with transparent bottoms (Costar, San Diego, CA, USA) and transfected with 2-5A (10 µM) with Lipofectamine 2000 or RL RNAs and Ctrl RNAs (2 µg/mL) using PolyJet reagent. At indicated times, caspase-3/7 activity in lysates was measured using the ApoONE homogenous caspase-3 and -7 assay kit (Promega, Madison, WI, USA) and normalized to control mock-treated cells. Experiments were performed in triplicate and shown as mean ± SD. In experiments expressing RIG I and DHX15 plasmids (50 ng/well), cells were transfected with RL RNAs and Ctrl RNAs (2 µg/mL) using PolyJet reagent after 16 h of plasmid expression.

### 2.11. RNA Isolation and Quantitative Real-Time PCR

Total RNA was isolated from cells using Trizol reagent (Invitrogen), as per the manufacturer instructions. Reverse transcription and cDNA synthesis was performed using random decamers and a RETROscript cDNA synthesis kit (Life Technologies; Thermo Fisher Scientific, Carlsbad, CA, USA). Gene expression was determined by quantitative reverse transcription polymerase chain reaction (qRT-PCR) using SYBR Green PCR Master Mix (Bio-Rad Laboratories Inc., Hercules, CA, USA) using gene-specific primers (Table 3) and normalized to GAPDH expression. For viruses from cell supernatants, 10 μL of RNA was directly processed for cDNA synthesis using random decamers and a RETROscript cDNA synthesis kit (Life Technologies; Thermo Fisher Scientific, Carlsbad, CA, USA). The viral RNA copy number was determined using the CVB3 standard curve as described above.

### 2.12. RNA Binding Assays

Cell lysates were prepared from DHX15 knockdown cells expressing Myc-DHX15 or mutant constructs in lysis buffer containing protease, phosphatase, and RNase inhibitors as described below. For poly(I-C) pulldown assays, Poly C agarose beads (Millipore Sigma, St. Louis, MO, USA) were rehydrated in nuclease-free water at 4 °C on a shaker for 10 min followed by resuspension in 2 volumes of Poly I beads (Millipore Sigma, St. Louis, MO, USA). The mixture was left on the shaker overnight at 4 °C. Beads were washed 3 times with wash buffer (50 mM Tris [pH 7.0], 150 mM NaCl) and resuspended as 50% slurry. For performing the pulldown assay, beads were equilibrated in the binding buffer (50 mM Tris [pH 7.5], 150 mM NaCl, 1 mM EDTA, 1% NP-40) as a 10% slurry and then combined with an equal volume of whole-cell extract that was prediluted to contain 3 μg of protein. The cell extracts were supplemented with protease and phosphatase inhibitors and 25 U of RNase inhibitor/mL. The mixture was incubated with gentle agitation for 1 h at 4 °C. Beads were centrifuged at 1000× *g*, rinsed three times with binding buffer, and then resuspended in 3 volumes of 1× sodium dodecyl sulfate polyacrylamide gel electrophoresis sample buffer, analyzed by sodium dodecyl sulfate polyacrylamide gel electrophoresis gels, and immunoblot analysis.

### 2.13. Western Blot Analysis

Cell pellets were lysed in NP-40 lysis buffer containing 0.5% NP-40, 90 mM KCl, 5 mM magnesium acetate, 20 mM Tris, pH 7.5, 5 mM β-mercaptoethanol, 0.1 M phenylmethylsulfonyl fluoride (PMSF), 0.2 mM sodium orthovanadate, 50 mM NaF, 10 mM glycerophosphate, and protease inhibitor (Roche Diagnostics, Indianapolis, IN, USA). The lysates were clarified by centrifugation at 12,000× *g* (4 °C for 15 min). For cell fractionation experiments, cells were lysed in buffer and separated into nuclear and cytoplasmic extracts using a nuclear/cytosol fractionation kit (MBL International Corporation, Woburn, MA, USA). Protein concentrations in the supernatants were determined by Bradford assay using bovine serum albumin as a standard. Proteins (15 to 50 μg or as indicated per lane) were separated in polyacrylamide gels containing SDS, transferred to nitrocellulose membranes (Bio-Rad, Hercules, CA, USA) or PVDF membrane (Bio-Rad, Hercules, CA, USA), and probed with different primary antibodies, according to the manufacturer’s protocols. The membranes were incubated with goat antimouse or goat antirabbit antibody tagged with horseradish peroxidase (Cell Signaling, Danvers, MA, USA), and immunoreactive bands were detected by enhanced chemiluminescence (Bio-Rad (Hercules, CA, USA )and Boston BioproductsAshland, MA, USA) For determining LC3-II/β-actin, cleaved PARP/β-actin, Phospho-JNK/Total-JNK, Phospho-P38/Total-P38, p65 nuclear translocation/Lamin, and IκBα degradation levels/β-Actin, the intensity of each band was determined by densitometry using Image J (Version 1.54) (National Institutes of Health), and the relative intensities were calculated by normalizing to β-actin from the corresponding samples. The ratios are graphically presented as the mean ± SEM from three independent experiments. Images were processed using Adobe Photoshop CS4 (Adobe, San Jose, CA, USA). In some instances, nonspecific lanes were cropped to generate the images, and the boundaries are indicated in the representative figures.

### 2.14. Immunofluorescence Analysis

Cells were cultured on glass coverslips, and after treatment, the cells were fixed with 4% paraformaldehyde (Boston Bioproducts) for 15 min and permeabilized with 0.1% Triton X-100 in phosphate-buffered saline (PBS) for 15 min. The cells were then blocked with 3% bovine serum albumin (BSA) for 1 h at room temperature, washed with PBS, and incubated for 1 h with NF-κB p65 subunit antibody (1:250). Alexa488-conjugated anti-immunoglobulin antibody (1:400) (Molecular Probes, Eugene, OR, USA)) was used as a secondary antibody. Cell nuclei were stained with Vectashield, with DAPI (4′,6-diamidino-2-phenylindole) to stain the nucleus (Vector Laboratories, Burlingame, CA, USA). Cells were imaged on a confocal fluorescence microscope, and the analysis and processing of images were performed using a Leica CS SP5 multiphoton laser scanning confocal microscope (Leica Microsystems, Wetzlar GmbH, Germany) and ImageJ software.

### 2.15. Coimmunoprecipitation

HT1080 cells were transfected with pkMyc vector or pkMyc DHX15 (5 μg/plate) plasmid, and after 24 h, cells were transfected with RL RNAs or Ctrl RNA (2 μg/mL) using PolyJet reagent or 2-5A (10 μM) using lipofectamine 2000 for 8 h. Cells were lysed in buffer containing 0.5% NP-40, 90 mM KCl, 5 mM magnesium acetate, 20 mM Tris (pH 7.5), 5 mM β mercaptoethanol, 0.1 M phenylmethylsulfonyl fluoride (PMSF), 0.2 mM sodium orthovanadate, 50 mM NaF, 10 mM glycerophosphate, and protease inhibitor (Roche Diagnostics, Indianapolis, IN) on ice for 45 min. Lysates were clarified by centrifugation at 12,000 rpm for 15 min at 4 °C. An amount of 500 μg of clarified cell lysates was precleared and mixed with control IgG or using Myc-tag beads and rotated end to end at 4 °C for 16 h. The beads were washed five times in NP40 lysis buffer. Samples were boiled in SDS sample buffer and immunoprecipitated proteins were analyzed by protein gel electrophoresis and immunoblotting using the indicated antibodies.

### 2.16. Luciferase Reporter Assay

Cells (8 × 10^4^) were seeded in a 12-well plate and transfected with the indicated luciferase reporter plasmids (0.5 μg/well), along with *Renilla* plasmid (50 ng/well) or β-galactosidase expressing plasmid (50 ng/well) and Rig-I or DHX15 expression plasmids (WT or mutants as indicated, 100 ng/well). After 24 h, cells were transfected with RL RNAs and Ctrl RNAs (2 µg/mL). Luciferase and β-gal activities were determined at the indicated times after transfection using the Dual Luciferase Assay kit (Promega; USA) or β-Galactosidase assay. Luminescence and absorbance were measured in 96-well opaque plates using a SpectraMax iD5 Microplate Reader (Molecular Devices). Luciferase activity was normalized to *Renilla* or β-gal values, and the fold induction was calculated relative to the mock transfected samples. The assay was performed in triplicates and plotted as mean ± SE from three independent experiments.

### 2.17. Statistical Analysis

All values are presented as mean ± SEM from at least three independent experiments or are representative of three independent experiments performed in triplicate and shown as mean ± SD. Student’s *t*-tests were used for determining statistical significance between groups using Prism8 (GraphPad, V10, Boston, MA, USA) software, and *p* < 0.05 was considered significant.

## 3. Results

### 3.1. DHX15 and Rig-I Are Required for Cell Death Induced by RNase L Activation

**Figure 1:**
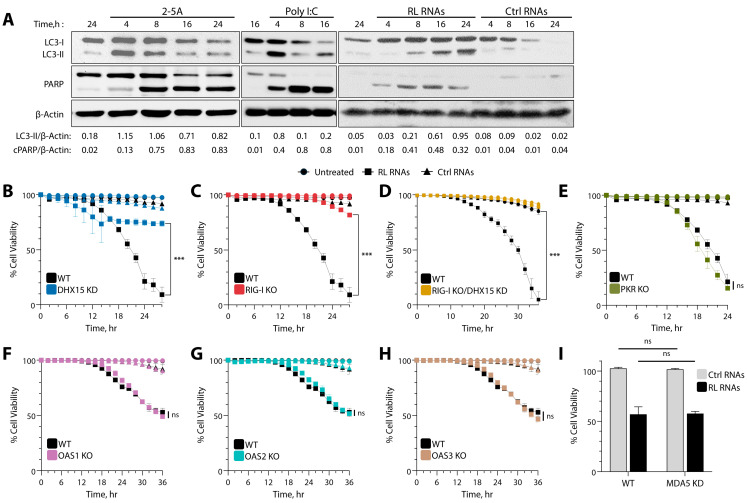
Role of dsRNA sensors in cell death induced by RNase L. (**A**) HT1080 cells were transfected with 2-5A (10 µM), Poly I:C (2 µg/mL), RL RNAs, or Ctrl RNAs (2 µg/mL) for indicated time points and the conversion of LC3-I to LC3-II. the cleavage of PARP was normalized to β-actin by immunoblotting, and band intensities were plotted as ratios. Kinetics of cell death induced by RL RNAs (2 µg/mL) were compared to Ctrl RNAs (2 µg/mL), and the percentage of the cell viability was determined by real-time imaging using dual dyes in WT HT1080 cells and compared to (**B**) DHX15 KD, (**C**) Rig-I KO, (**D**) Rig-I KO/DHX15 KD, (**E**) PKR KO, (**F**) OAS1 KO, (**G**) OAS2 KO, and (**H**) OAS3 KO cells. The percentage of cell survival in each well was determined by quantitating dead cells and normalized to the total number of cells at each time point. Data are representative of four values per well performed in triplicate and shown as mean ± SD. (**I**) HT1080 cells were transfected with control or MDA5 siRNA (5 nM) and, after 24 h, treated with RL RNAs or Ctrl RNAs (2 µg/mL)thepercentage of the cell viability was determined by MTT assay. The results are representative of three independent experiments performed in triplicate ± SD. WT: wild-type; RL RNAs: RNase L-cleaved small RNAs; Ctrl RNAs: control small RNAs; *** *p* < 0.001; ns: not significant.

To study the role of RNase L activation on cell death, human fibrosarcoma HT1080 cells that have been widely used as a model cell line to study IFN signaling pathways were transfected with 2-5A to specifically activate RNase L or synthetic dsRNA polyI:C to produce 2-5A in cells. In line with our previous findings, we observed the early activation of autophagy, as determined by the lipidation of LC3 and an increase in the cleavage of PARP, a hallmark of apoptosis, later in time, up to 24 h (Figure 1A) [59]. Further, the transfection of RNase L-cleaved RNAs (RL RNAs), purified as <200 nt RNAs from cells following RNase L activation (described in methods) but not control RNAs into HT1080 cells, resulted in similar LC3 lipidation and the cleavage of PARP (Figure 1A) and were used to study the signaling pathways that promote cell death by RNase L-cleaved RNAs. To determine whether any of the specific sensors in the dsRNA signaling pathways in the antiviral system might affect cell death in response to RNase L activity, we generated HT1080 cells lacking the expression of *Rig-I*, *PKR*, *OAS1*, *OAS2*, or *OAS3* genes using CRISPR/Cas9 technology, or lentivirus-mediated DHX15 knockdown cells (DHX15 KD) and *Rig-I KO*/DHX15 KD cells (Figure S1A,B). Parental and dsRNA signaling pathway sensor ablated cells were treated with RL RNAs and Ctrl RNAs, and cell survival was monitored in real time using dual dyes and an IncuCyte Live-Cell imaging system. Treatment of the WT cells with RL RNAs resulted in significant cell death, while cells treated with control RNAs remained viable over a period of time. Compared to WT cells, cells lacking Rig-I expression or knockdown for DHX15-promoted cell survival in response to RL RNA transfection, while the control RNAs had no effect, suggesting that both Rig-I and DHX15 are required for cell death. (Figure 1B,C). Depleting the cells of both Rig-I and DHX15 further increased cell viability after RL RNA treatment than the single gene ablated cells (Figure 1D). In contrast, the depletion of other dsRNA sensors, like PKR, OAS1, OAS2, or OAS3, or the knockdown of MDA5 by siRNA resulted in cell death comparable to WT cells, suggesting that these proteins are dispensable for cell death induction by RL RNAs (Figure 1E–I). These results suggest a critical role of DHX15 and Rig-I in cell death induced by RNase L activation.

### 3.2. DHX15 and Rig-I Mediate Apoptotic Cell Death by RNase L Cleavage Products

**Figure 2:**
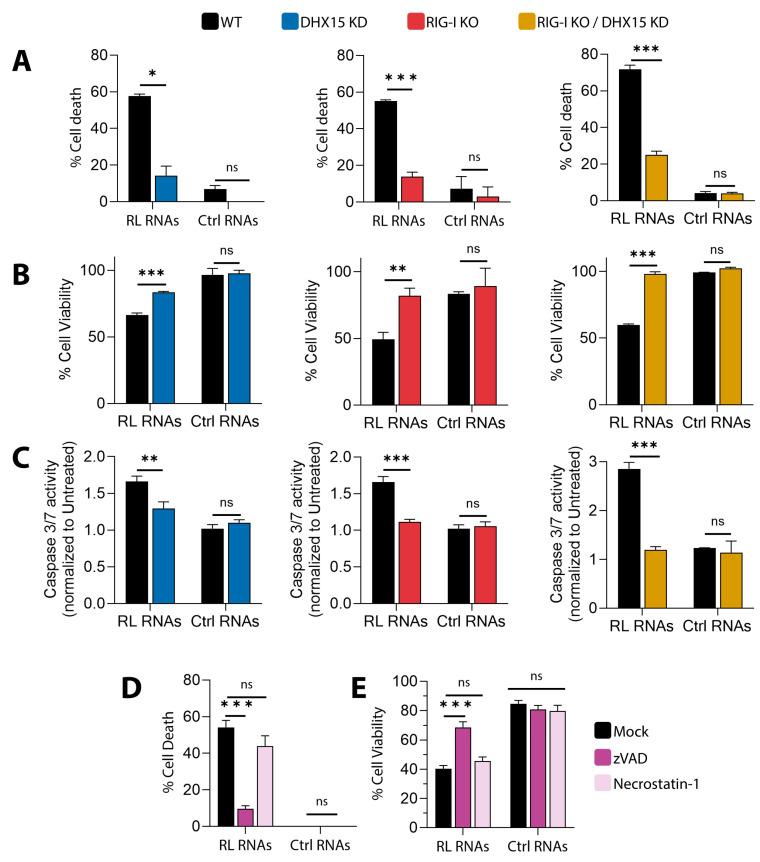
RL RNAs signal through DHX15 and Rig-I to induce apoptotic cell death. HT1080 WT, DHX15 KD, Rig-I KO, or Rig-I KO/DHX15 KD cells were transfected with RL RNAs or Ctrl RNAs (2 µg/mL). (**A**) The percentage of cell death by trypan blue exclusion, (**B**) the percentage of cell viability by MTT assay, and (**C**) caspase-3/7 activity were determined. WT cells were mock-treated, pretreated with zVAD-FMK (20 μΜ) or necrostatin-1 (20 μΜ), and transfected after 1 h with RL RNAs or Ctrl RNAs (2 µg/mL). (**D**) The percentage of cell death by trypan blue exclusion and (**E**) the percentage of cell viability by MTT assay were determined. The results are representative of three independent experiments performed in triplicate ± SD. KO: knockout; KD: knockdown; WT: wild-type; RL RNAs: RNase L-cleaved small RNAs; Ctrl RNAs: control small RNAs; * *p* < 0.05; ** *p* < 0.01; *** *p* < 0.001; ns: not significant.

To investigate the role of DHX15 and Rig-I in cell death by RNase L, we established stable HT1080 cells that expressed lentiviral-mediated shRNA to knockdown DHX15, Rig-I knockout, and in combination (Rig-I KO/DHX15KD). The knockdown efficiency of DHX15 using various targeting shRNAs was determined on immunoblots and compared with increased cell viability on treatment with RL RNAs (Figure S2). Cells expressing LV1 shRNA, targeting the 3′UTR of DHX15, showed the significant knockdown of DHX15, which correlated with increased viability in response to RL RNAs and were used in this study. These cell lines and parental HT1080 cells were analyzed for cell death in response to RL RNA transfection and compared to control RNAs by monitoring cell death using trypan blue exclusion assay (Figure 2A) and the loss of cell viability using MTT cell proliferation assay (Figure 2B). Cells lacking Rig-I or the knockdown of DHX15 or both showed reduced cell death, while WT cells treated with RL RNAs caused significant loss of cell viability in both assays. To characterize the pathways promoting RL-mediated cell death involving Rig-I and DHX15, we measured the enzyme activity of caspase-3/7, a hallmark of apoptotic cells using a fluorescent caspase-3/7 substrate (Figure 2C). Caspase-3 activation by RL RNAs required Rig-I and DHX15, as cells lacking either proteins or both showed reduced activity that correlated with increased survival. Similar increase in cell death, loss of viability, the activation of caspase-3, and the cleavage of PARP on immunoblots were observed when human primary Newborn Foreskin Fibroblasts (NuFF) were transfected with RL RNAs (Figure S3). Cells treated with pan-caspase inhibitor z-VAD-fmk (z-VAD) and not with necrostatin-1 rescued RL-induced cell death consistent with the activation of cell death by apoptosis (Figure 2D). These results show that RL RNAs induce cell death by apoptosis, and both Rig-I and DHX15 are required for this effect.

### 3.3. RNase L Signals via DHX15 and Rig-I to Promote MAVS-Dependent Apoptosis

**Figure 3:**
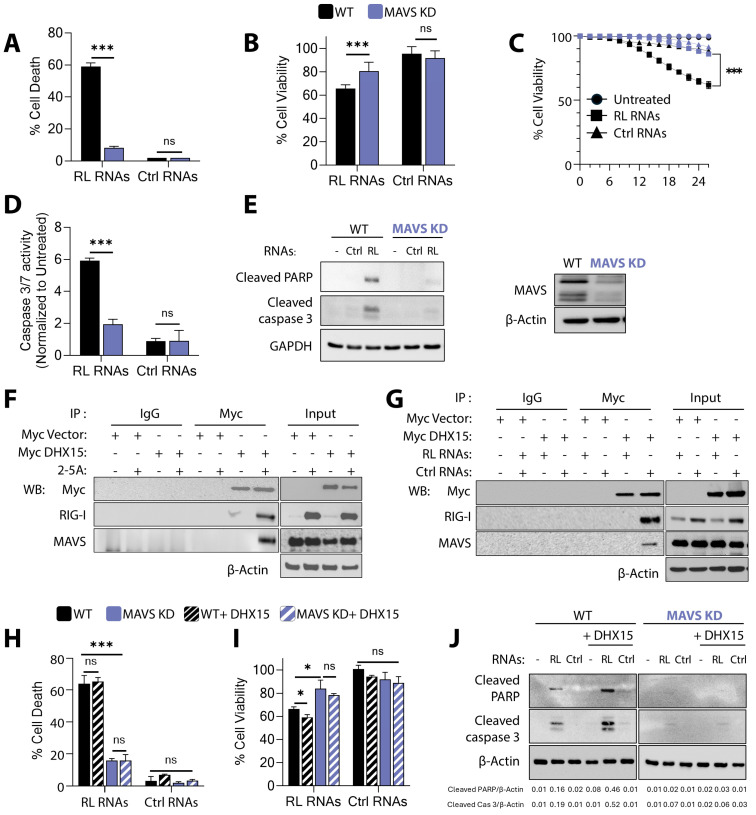
MAVS is required for RL RNA-mediated apoptosis. HT1080 WT and MAVS KD cells were transfected with RL RNAs or Ctrl RNAs (2 µg/mL). (**A**) The percentage of cell death by trypan blue exclusion, (**B**) the percentage of cell viability by MTT assay, (**C**) Kinetics of cell death in real time, and the percentage of cell viability were estimated. (**D**) Caspase-3/7 activity was determined. (**E**) Cell lysates were analyzed for cleaved PARP, cleaved caspase-3, and MAVS by immunoblotting. HT1080 cells expressing Myc vector or Myc-DHX15 were transfected with (**F**) 2-5A (10 µM) or (**G**) RL RNAs and Ctrl RNAs (2 µg/mL). Cell lysates were immunoprecipitated using anti-Myc or isotype control IgG, and the presence of Rig-I and MAVS analyzed by immunoblotting. The expression of the proteins in total cell lysates was analyzed using specific antibodies and normalized to β-actin levels. WT and MAVS KD cells overexpressing DHX15 or not were transfected with RL RNAs or Ctrl RNAs (2 µg/mL), and (**H**) the percentage of cell death by trypan blue exclusion, (**I**) the percentage of cell viability by MTT assay, and (**J**) the cleaved PARP and cleaved caspase-3 on immunoblots were determined. The band intensities were plotted as ratios. The representative immunoblots of experiments performed in duplicate are shown. The results are representative of three independent experiments performed in triplicate ± SD. KO: knockout; KD: knockdown; WT: wild-type; RL RNAs: RNase L-cleaved small RNAs; Ctrl RNAs: control small RNAs; * *p* < 0.05; *** *p* < 0.001; ns: not significant.

Rig-I, MDA5, and members of DExD/H-box RNA helicases including DHX15 that recognize dsRNA and these receptors signal via MAVS which isa critical downstream adaptor in RLR signaling that drives the innate response to virus infection. To determine the role of MAVS in apoptosis by RNase L-induced RL RNAs, we first established the stable knockdown of MAVS (MAVS KD) in HT1080 cells by lentiviral-mediated shRNA. Cell death and the loss of cell viability was monitored in WT and MAVS KD cells transfected with RL RNAs and compared to control RNAs. MAVS KD cells were highly resistant to cell death by RL RNAs, while WT cells were highly susceptible (Figure 3A,B). Cell survival in MAVS KD cells was determined in real time and compared to WT cells. The quantitative measurement of cell death after 24 h showed that 40% of WT cells were dead compared to 15% of MAVS KD cells (Figure 3C). Caspase-3/7 activation was monitored as a marker of apoptosis in RL RNA-treated cells, and MAVS KD cells showed 3-fold reduced enzyme activity compared to WT cells (Figure 3D). Compared to WT cells, no cleavage of PARP or caspase-3 by RL RNAs was observed on immunoblots (Figure 3E). Taken together, these further support the finding that MAVS is required for apoptosis by RL RNAs mediated by DHX15 and Rig-I.

Next, to determine the role of MAVS in RL RNA signaling, we first investigated interaction with DHX15 and Rig-I in response to RNase L activation by its unique ligand 2-5A. The treatment of HT1080 cells with 2-5A causes dimerization and the activation of the latent ribonuclease, RNase L, resulting in the degradation of diverse RNA substrates producing intracellular RL RNAs. Coimmunoprecipitation experiments revealed that DHX15 interacted with Rig-I and MAVS only in 2-5A-treated cells compared to mock-treated cells in which Rig-I levels are low and present in autoinhibited state (Figure 3F). To further confirm that the interaction was promoted by RL RNAs, DHX15-Rig-I-MAVS interaction was tested by coimmunoprecipitation in cells transfected by RL RNAs. Similar to 2-5A treatment, DHX15 interacted with Rig-I and MAVS only in the presence of RL RNAs and not control RNAs (Figure 3G). These results suggest that DHX15 interaction with Rig-I is induced by RL signaling when Rig-I levels are increased and promote association with MAVS.

Rig-I, on binding dsRNA and activation, oligomerizes with MAVS to transduce RLR signaling, and MAVS is required for signaling [11,14,68]. To define the epistatic relationship between DHX15 and MAVS, we used MAVS KD cells and evaluated signaling by RL RNAs by overexpressing DHX15. The overexpression of DHX15 did not increase cell death in MAVS KD cells with RL RNAs, and no significant change in the cleavage of PARP or caspase-3 was observed on immunoblots (Figure 3H–J). These results suggest that RL RNAs promote apoptosis via DHX15 and Rig-I at or above the level of MAVS, and apoptosis signals are relayed downstream via MAVS.

### 3.4. MAPK Signaling Induced by RNase L-Cleaved RNAs Is Required for DHX15-Rig-I-MAVS-Mediated Apoptosis

**Figure 4:**
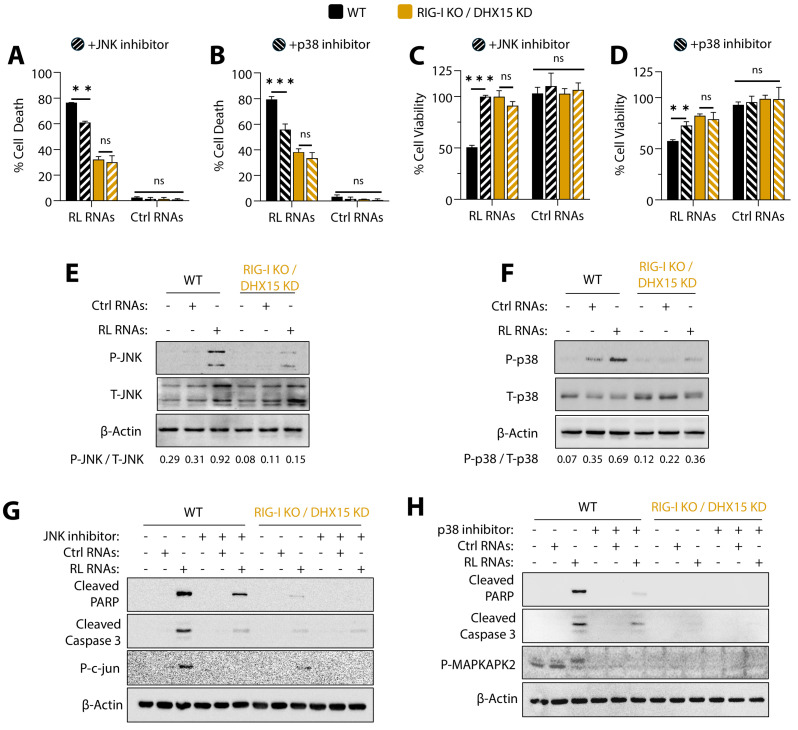
RL RNAs induce JNK and p38 phosphorylation and cell death: HT1080 WT and Rig-I KO/DHX15 KD cells were transfected with RL RNAs or Ctrl RNAs (2 µg/mL) in the absence or presence of the JNK inhibitor or p38 inhibitor as indicated. (**A**,**B**) The percentage of cell death by trypan blue exclusion and (**C**,**D**) cell viability by MTT assay were determined. Cell lysates were analyzed for the phosphorylation of JNK (**E**) and p38 (**F**) and normalized to the level of total JNK and p38, respectively, on immunoblots. Cell lysates from WT and Rig-I KO/DHX15 KD cells treated as above were analyzed on immunoblots for cleaved PARP, cleaved caspase-3, and phospho-c-Jun (**G**) or phospho-MAPKAPK2 (**H**) and normalized to β-actin levels. The representative immunoblots of experiments performed in duplicate are shown. The results are representative of three independent experiments performed in triplicate ± SD. KO: knockout; KD: knockdown; WT: wild-type; RL RNAs: RNase L-cleaved small RNAs; Ctrl RNAs: control small RNAs; ** *p* < 0.01; *** *p* < 0.001; ns: not significant.

Previously, we and others reported that RNase L activation by its ligand 2-5A or dsRNA and the treatment of cells with RL RNAs induced JNK and p38 signaling and sustained activation-promoted apoptosis [56,58,59,60,63,69]; DHX15 is reported to activate MAPK, IRF-3, and NF-kB in response to polyI:C and upon reovirus infection [39,40]. The overlap in the involvement of signaling pathways prompted us to test its activation and role in apoptosis by RL RNAs. To determine the roles of JNK and p38 in RL-induced apoptosis by DHX15 and Rig-I, WT and Rig-I KO/DHX15KD cells were transfected with RL or control RNAs in the absence or presence of JNK inhibitor (JNKi) or p38 inhibitor (p38i), and cell death and viability were measured. The inhibition of either JNK or p38 with specific pharmacological inhibitors resulted in greater survival in WT cells, while cells lacking both Rig-I and DHX15 were minimally impacted (Figure 4A–D). Both JNK and p38 were activated by phosphorylation in response to RL RNAs and not control RNAs in WT cells, while Rig-I KO/DHX15KD cells showed significantly decreased phosphorylation (Figure 4E,F). The increased cleavage of PARP and caspase-3 by RL RNAs was accordingly reduced by JNKi or p38i in WT cells, while the cleavage of apoptotic markers was barely detectable in Rig-I KO/DHX15KD cells even in the absence of the inhibitors (Figure 4G,H). The small amount of cleavage of the apoptotic markers with the individual inhibitors could possibly be due to the residual activity of the unaffected MAPK. These data support our observations that DHX15 and Rig-I are important for activating JNK and p38 for MAVS-mediated apoptosis by RL RNAs.

### 3.5. Apoptosis Induced by RNase L-Cleaved RNAs Is Independent of IRF-3 and NF-κB Signaling

**Figure 5:**
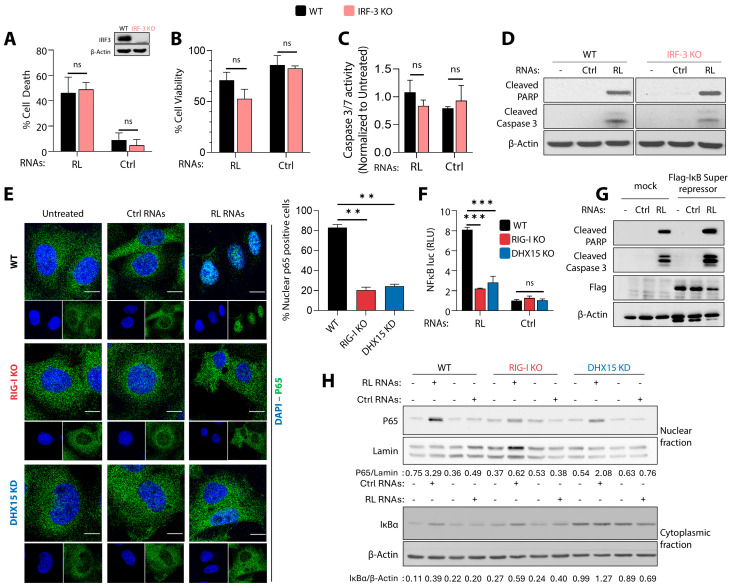
RL RNA-mediated apoptosis is independent of IRF-3 and NF-kB signaling. HT1080 WT and IRF-3 KO cells were transfected with RL RNAs or Ctrl RNAs (2 µg/mL). (**A**) The percentage of cell death by trypan blue exclusion, (**B**) the percentage of cell viability by MTT assay, and (**C**) caspase-3/7 activity were determined. The inset in (**A**) shows immunoblot of WT and IRF-3 KO cells for IRF-3 levels. (**D**) Cell lysates were analyzed for cleaved PARP and cleaved caspase-3 on immunoblots and normalized to β-actin levels. WT, Rig-I KO, and DHX15KD were (**E**) transfected with RL RNAs or Ctrl RNAs (2 µg/mL) for 8 h, and the nuclear translocation of the NF-kB p65 subunit (green) was determined by immunofluorescence; nuclei were stained with DAPI. The representative images of cells were visualized under a confocal microscope at 60×. The scale bar represents 50 μm. The percentage of cells showing nuclear p65 cells were quantified in 3 random fields. At least 100 cells were quantified. The data represent mean ± SD; (**F**) co-transfected with NF-kB-luc reporter plasmids and *Renilla*-luc plasmid. After 24 h, the cells were treated with RL RNAs or Ctrl RNAs (2 µg/mL), and 8 h later, luciferase activity was measured and normalized to the levels of *Renilla* luciferase and shown as fold induction. Results are representative of three independent experiments performed in triplicate ± SD. (**G**) HT1080 WT cells were transfected with FLAG-IκB super-repressor and, 24 h later, with RL RNAs or Ctrl RNAs (2 µg/mL). Cell lysates were analyzed for cleaved PARP, cleaved caspase-3, and FLAG-IkB on immunoblots and normalized to β-actin levels. (**H**) WT, DHX15 KD, and RIG I KO cells were transfected with RL RNAs or Ctrl RNAs (2 µg/mL) for 8 h. Cell lysates were fractionated into nuclear and cytosol fractions and analyzed by immunoblotting using indicated antibodies. The levels of NF-κB p65 subunit in the nuclear extract normalized to Lamin or the degradation of IκBα in cytosol extract normalized to β-actin levels was determined by Image J analysis. The representative immunoblots of experiments performed in duplicate are shown. KO: knockout; WT: wild-type; RL RNAs: RNase L-cleaved small RNAs; Ctrl RNAs: control small RNAs; ** *p* < 0.01; *** *p* < 0.001; ns: not significant.

During virus infection, dsRNA replication intermediates are sensed by cytosolic Rig-like receptors and activate IRF-3 and NF-kB transcription factors to upregulate IFN, cytokines, and antiviral factors that can induce apoptosis [3]. Furthermore, Rig-I-mediated IRF-3 activation by dsRNA induces apoptosis by a distinct mechanism that is independent of its role as a transcription factor [29]. NF-kB transcriptionally induces proinflammatory cytokines and regulates cell survival [70]. We have shown previously that RNase L activates IRF-3 and NF-kB to produce type I IFN; however, the role of IRF-3 and NF-kB in inducing apoptosis by RNase L is not known [62]. To determine the role of IRF-3 in apoptosis by RL RNAs, cell death, cell viability, and the induction of caspase-3/7 activity in WT and *IRF-3* KO (deleted by CRISPR/Cas9) cells were compared to cells treated with control RNAs. No statistically significant difference in any of the apoptosis assays was observed in cells ablated for IRF-3 (Figure 5A–C). This observation was supported by the comparable cleavage of PARP, anda small difference in caspase-3 cleavage on immunoblots in both WT and IRF-3 KO cells (Figure 5D). These results suggest that the apoptosis induced by RNase L-cleaved RL RNAs is independent of IRF-3.

Multiple signals that activate NF-κB by phosphorylation and ubiquitination result in the degradation of IκB and the translocation of phospho-NF-κB p65 to the nucleus, where it binds κB sites in the promoter region of target genes and promotes transcription [71]. To test the role of Rig--I and DHX15 in the activation of NF-κB, we first monitored the activation of NF-κB by the translocation of the p65 subunit to the nucleus. We then determined the role of DHX15 and Rig-I in NF-kB promoter activation using luciferase reporter constructs in WT, Rig-I KO, and DHX15 KD cells transfected with RL RNAs and compared to control RNAs. In response to RL RNAs, the translocation of p65 to the nucleus and the corresponding activity of the NF-kB promoter were significantly reduced in both DHX15 KD and Rig-I KO cells compared to WT cells, while control RNAs did not contribute to NF-kB activities. (Figure 5E,F). We expressed the IκB super repressor, which inhibits the activation of NF-κB in WT cells and monitored the activation of apoptosis markers by immunoblotting. We found no significant difference in the cleavage of PARP and caspase-3, suggesting that the activation of NF-κB or the target genes is not required for the induction of apoptosis by RL RNAs (Figure 5G). Compared to WT cells treated with RL RNAs, cells lacking Rig-I or DHX15 KD cells show a reduced accumulation of p65 in nuclear fraction (Figure 5H). A decrease in nuclear p65 correlated with a decrease in cytosolic Iκb degradation, suggesting that DHX15 and Rig-I are required for optimal NF-κB activation.

### 3.6. Induction of IFN and Proinflammatory Cytokines by RNase L-Cleaved RNAs Is Dependent on DHX15 and Rig-I

**Figure 6:**
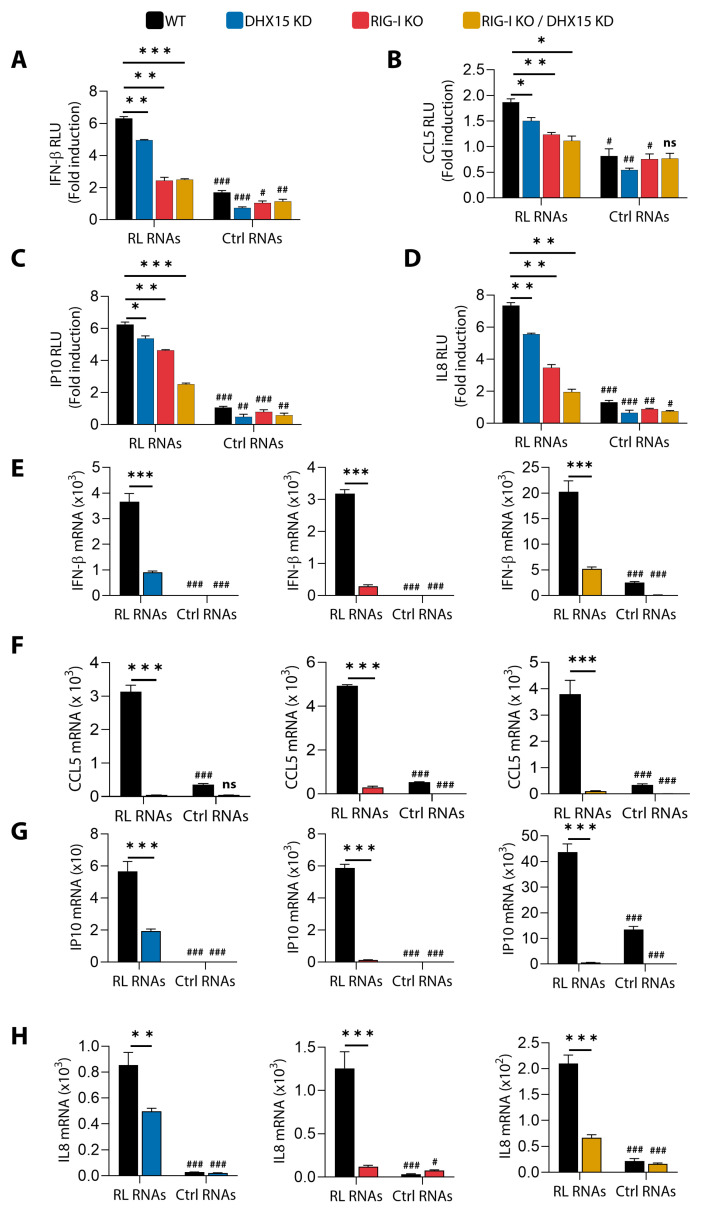
IFN and cytokine induction by RL RNA is mediated by DHX15 and Rig-I. HT1080 WT, Rig-I KO, DHX15KD, and Rig-I KO/DHX15KD cells were transfected with (**A**) IFN-β-luc, (**B**) CCL5-luc, (**C**) IP-10-luc, and (**D**) IL-8-luc reporter plasmids along with *Renilla* luc or β-galactosidase plasmids. After 24 h, the cells were treated with RL RNAs or Ctrl RNAs (2 µg/mL), and 8 h later, luciferase activity was measured and normalized to levels of *Renilla* luciferase or β-galactosidase levels and shown as fold induction. HT1080 WT, Rig-I KO, DHX15KD, and Rig-I KO/DHX15KD cells were transfected with RL RNAs or Ctrl RNAs (2 µg/mL), and 8 h later, (**E**) IFN-β, (**F**) CCL5, (**G**) IP-10, and (**H**) IL-8 mRNA levels were measured by qRT-PCR and normalized to GAPDH mRNA levels. Induction by RL RNAs in the various genotypes was compared to WT and significance shown as *. Induction by RL RNAs compared to Ctrl RNAs within a cell type is indicated by #. The data represent mean ± SD performed in triplicates. WT: wild-type, KO: knockout; KD: knockdown; RL RNAs: RNase L-cleaved small RNAs; Ctrl RNAs: control small RNAs; * *p* < 0.05, # *p* < 0.05; ** *p* < 0.01, ## *p* < 0.01; *** *p* < 0.001, ### *p* < 0.001; ns: not significant.

To investigate the role of DHX15 and Rig-I in inducing IFN and proinflammatory cytokines, we monitored IFNβ, CCL5 (RANTES), IP-10, or interleukin 8 (IL-8) promoter activation using luciferase reporter constructs in WT, DHX15 KD, Rig-I KO, and Rig-I KO/DHX15 KD cells by introducing RL RNAs and comparing them to control RNAs. The RL RNA induction of IFNβ, CCL5, IP-10, and IL-8 promoters was reduced in cells lacking either DHX15 or Rig-I, and the promoter activity levels were further reduced in cells lacking both Rig-I and DHX15 (Figure 6A–D). Similar decreases in mRNA levels of *IFNβ, CCL5, IP-10*, and *IL-8* were observed in response to RL RNAs in cells depleted of Rig-I or DHX15 compared to WT cells, as determined by real-time PCR analysis. Additionally, cells lacking both proteins showed a significantly lower induction of mRNA of *IFNβ* and proinflammatory cytokines (Figure 6E–H). Taken together, our results suggest that DHX15 and Rig-I are required for the RL RNA-mediated activation of IRF-3 and NF-kB to produce IFNβ and proinflammatory cytokines; however, the induction of apoptosis by RL RNAs is distinct and independent of IRF-3 and NF-kB signaling.

### 3.7. RNA Binding by DHX15 and Rig-I Triggers RNase L-Mediated Apoptosis

**Figure 7:**
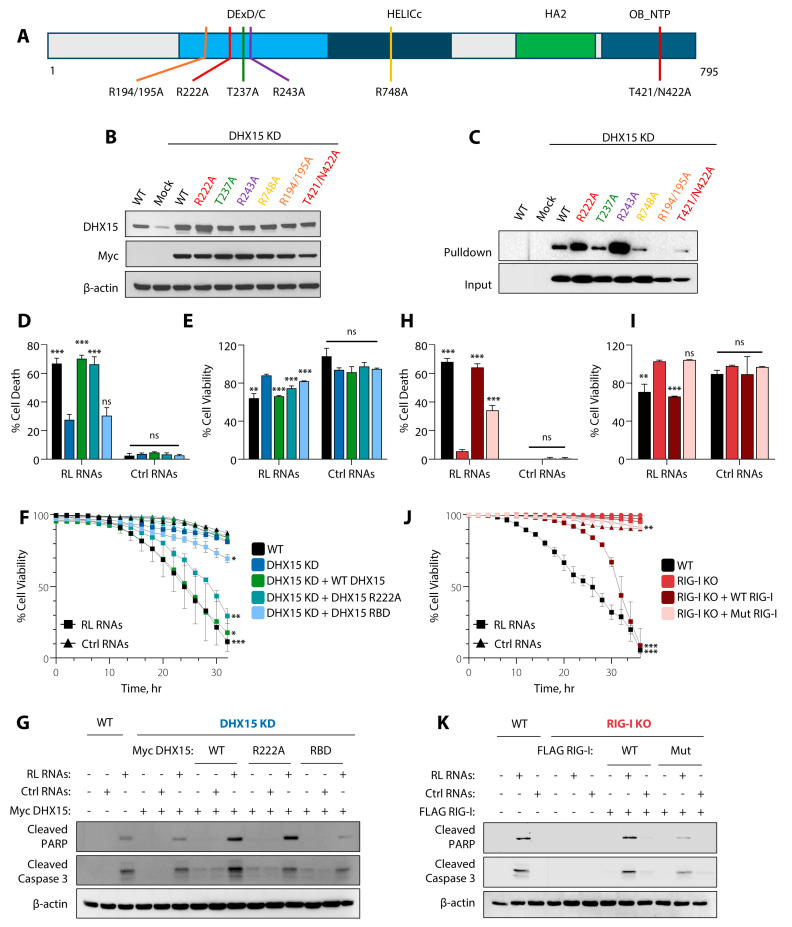
Effect of DHX15 and Rig-I mutations on apoptosis. (**A**) Schematic presentation of the domain structure of DHX15 and sites of point mutations. DExD/C: DEAD-like helicase superfamily domain; HELICc: helicase superfamily C-terminal domain: HA2: C-terminal helicase-associated domain; OB_NTP: oligonucleotide binding fold domain. (**B**) The expression of DHX15 WT and mutant plasmids in DHX15KD cells was analyzed by immunoblotting using indicated antibodies. ℜ-Actin was used as the loading control. (**C**) DHX15KD cells were transfected with empty vector (mock) or the indicated Myc-DHX15 plasmid constructs, and cell lysates were incubated with polyI:C-agarose beads and pulled-down DHX15 proteins detected by immunoblotting. Immunoblots without pulldown show the input levels of the Myc-DHX15 proteins. HT1080 WT and DHX15 KD cells expressing the indicated DHX15 plasmids were transfected with RL RNAs or Ctrl RNAs (2 µg/mL). (**D**) The percentage of cell death by trypan blue exclusion, (**E**) the percentage of cell viability by MTT assay, (**F**) Kinetics of cell death in real time, and the percentage of cell viability were estimated. The data represent mean ± SD performed in triplicates. (**G**) Cell lysates were analyzed for cleaved PARP and cleaved caspase-3 on immunoblots and normalized to b-actin levels. HT1080 WT and Rig-I KO cells expressing the indicated Rig-I plasmids were transfected with RL RNAs or Ctrl RNAs (2 µg/mL). (**H**) The percentage of cell death by trypan blue exclusion, (**I**) the percentage of cell viability by MTT assay, (**J**) Kinetics of cell death in real time, and the percentage of cell viability were estimated. The data represent mean ± SD performed in triplicates. (**K**) Cell lysates were analyzed for cleaved PARP and cleaved caspase-3 on immunoblots and normalized to β-actin levels. The representative immunoblots of experiments performed in duplicate are shown. WT: wild-type, Mut: mutant, KO: knockout; KD: knockdown; RL RNAs: RNase L-cleaved small RNAs; Ctrl RNAs: control small RNAs; * *p* < 0.05; ** *p* < 0.01; *** *p* < 0.001; ns: not significant.

Both DHX15 and Rig-I are RNA helicases; however, the biochemical features that mediate apoptosis by RL RNAs are not known. Structural and biochemical studies have identified domains and amino acid residues in Rig-I that are critical for activation and signaling in response to diverse dsRNA substrates [21,22,72]. In this study, we have used Rig-I RNA binding mutant (Rig-I K858/861A, Mut), characterized in other studies as defective in innate immune signaling, and analyzed apoptosis induction by RL RNAs [73]. To determine the features of DHX15, we generated point mutants targeting amino acid residues that are predicted to form an RNA binding pocket based on DHX15 crystal structure [74] and R222 residue that is mutated in AML patients and needed for splicing [75] (Figure 7A). The Myc-tagged DHX15 mutant constructs were expressed in DHX15 KD cells, and no significant difference in the expression levels was observed compared to endogenous or Myc-DHX15 WT expression levels on immunoblots (Figure 7B). We assessed the ability of WT and mutant DHX15 proteins binding to dsRNA by polyI:C RNA pulldown assays. The mutant DHX15 proteins bound to dsRNA to variable levels and a comparison of the binding with dsRNA in pulldown assays shows the requirement of R194/R195 residues for binding RNA, as no protein was detected that bound to dsRNA, while R222A and R243A bound to dsRNA more strongly compared to WT. DHX15 T421/N422A showed reduced dsRNA binding compared to WT and may contribute to binding RNA (Figure 7C). The data show that DHX15 R194/195A is defective in dsRNA binding and was used in our experiments.

To test the role of DHX15 RNA binding in cell death, DHX15 WT, R194/195A, or R222A mutants were expressed in DHX15 KD cells, and the cell death, viability, and kinetics of cell death by RL RNA and control RNA were determined. The expression of the RNA binding defective mutant, R194/195A (RBD), did not rescue cell death, while R222A showed similar levels of loss of viability, like WT DHX15 (Figure 7D–F). Additionally, the expression of DHX15 WT and R222A caused the increased cleavage of PARP and caspase-3 in DHX15 KD cells, while the DHX15 RBD mutant showed reduced cleavage comparable to DHX15 KD cells (Figure 7G). We tested the requirement of Rig-I RNA binding in Rig-I KO cells expressing WT or RNA binding mutant (Mut) in promoting cell death. The expression of WT Rig-I restored cell death in Rig-I KO cells, while the RNA binding defective mutant showed significantly reduced cell death (Figure 7H–J). An increase in the cleavage of PARP and caspase-3 was observed in Rig-KO cells expressing WT Rig-I compared to RBD mutant (Figure 7K).

**Figure 8:**
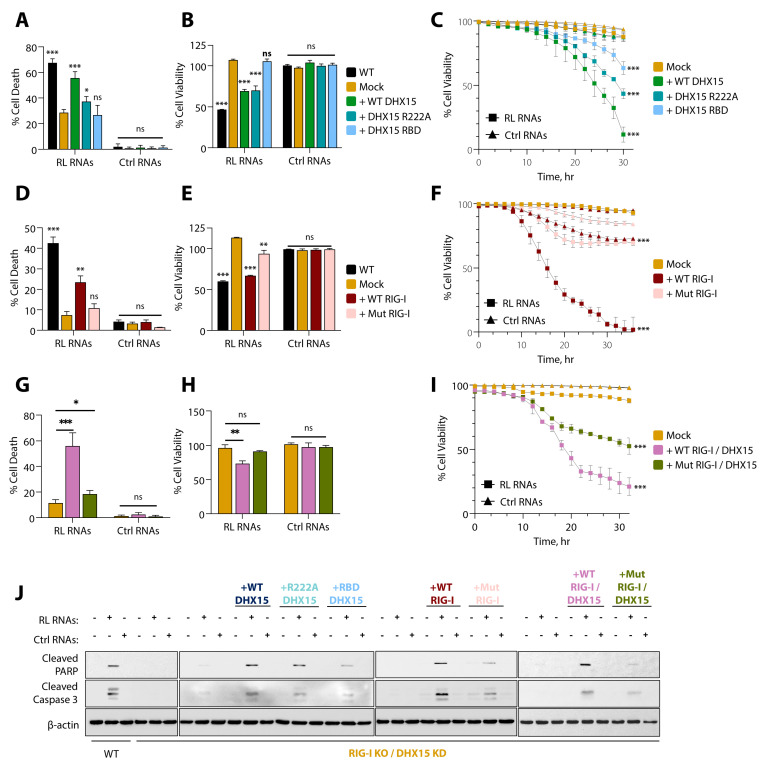
RNA binding defective mutants of DHX15 and Rig-I promote cell viability in response to RL RNAs. Rig-I KO/DHX15 KD cells expressing the indicated DHX15, Rig-I, or both DHX15 and Rig-I plasmids were transfected with RL RNAs or Ctrl RNAs (2 µg/mL). (**A**,**D**,**G**) The percentage of cell death by trypan blue exclusion, (**B**,**E**,**H**) the percentage of cell viability by MTT assay, (**C**,**F**,**I**) the kinetics of cell death in real time, and the percentage of cell viability were estimated. The data represent mean ± SD performed in triplicates. (**J**) Cell lysates were analyzed for cleaved PARP and leaved caspase-3 on immunoblots and normalized to β-actin levels. The representative immunoblots of experiments performed in duplicate are shown. Mut: RNA binding mutant, WT: wild-type, KO: knockout; KD: knockdown; RL RNAs: RNase L-cleaved small RNAs; Ctrl RNAs: control small RNAs; * *p* < 0.05; ** *p* < 0.01; *** *p* < 0.001; ns: not significant.

We then tested if the expression of either DHX15 or Rig-I alone is sufficient to induce cell death by expressing WT and RBD mutant proteins in Rig-I KO/DHX15 KD (dKO) cells and compared cell death and viability. The expression of WT DHX15 in dKO cells increased cell death compared to the RBD mutant; however, the levels were lower than the HT1080 WT cells (Figure 8A–C). The expression of WT Rig-I was also more effective at inducing cell death compared to the RBD mutant (Figure 8D–F). To test whether RNA binding by both DHX15 and Rig-I was required to induce cell death by RL RNAs, dKO cells were transfected with Rig-I and DHX15 WT constructs or Rig-I and DHX15 RBD mutants, and cell death and viability by RL RNAs and control RNAs were determined. Compared to cells expressing both WT proteins, cells expressing the RBD mutants of both Rig-I and DHX15 showed less cell death and increased cell viability (Figure 8G–I). Accordingly, reduced cleavage of PARP and caspase-3 was observed in dKO cells expressing the RBD mutants of both DHX15 and Rig-I (Figure 8J). These results show that RNA binding by both DHX15 and Rig-I is required for apoptosis by RNase L-cleaved RNAs.

### 3.8. Apoptosis Induced by RNase L Impacts CVB3 Pathogenesis

**Figure 9:**
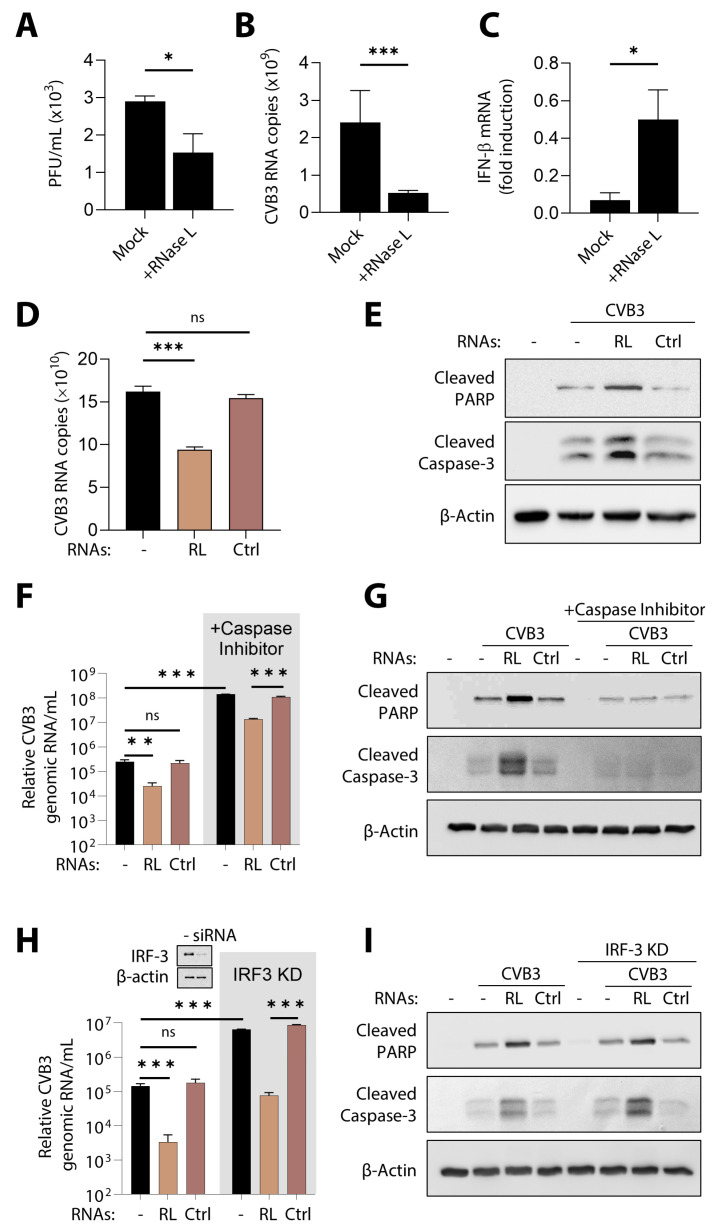
Apoptosis by RL RNAs enhances RNase L antiviral effect during CVB3 infection. HeLa-M cells (mock) or stably expressing WT RNase L were infected with CVB3 (MOI = 0.1) for 24 h. (**A**) Viral yields were determined by plaque assay, (**B**) the copy numbers of CVB3 genomic RNA were determined by real-time RT-PCR as described under methods, and (**C**) IFN-β mRNA levels in CVB3-infected cells were measured by qRT-PCR, normalized to GAPDH mRNA levels, and shown as fold induction. The data represent mean ± SD performed in triplicates. HeLa-M cells were transfected with RL RNAs or Ctrl RNAs (2 µg/mL) and infected with CVB3 (MOI 0.1), and after 24 h, (**D**) the copy numbers of CVB3 genomic RNA were determined by real-time RT-PCR. The data represent mean ± SD performed in triplicates. (**E**) Cell lysates were analyzed for cleaved PARP and cleaved caspase-3 on immunoblots and normalized to β-actin levels. (**F**) HeLa-M cells were transfected with RL RNAs or Ctrl RNAs (2 µg/mL) and infected with CVB3 (MOI = 1.0) with or without Ac-DEVD-CHO (20 mM), and after 24 h, the copy numbers of CVB3 genomic RNA were determined by real-time RT-PCR. The data represent mean ± SD performed in triplicates, and (**G**) cell lysates were analyzed for cleaved PARP and cleaved caspase-3 on immunoblots and normalized to β-actin levels. HeLa-M cells were transfected with control or siRNA specific for IRF-3 (50 mM) and, after 36 h, transfected with RL RNAs or Ctrl RNAs (2 µg/mL) and infected with CVB3 (MOI = 1.0). (**H**) The copy numbers of CVB3 genomic RNA were determined by real-time RT-PCR. The inset shows the immunoblot of WT and siIRF-3 cells for IRF-3 levels. The data represent mean ± SD performed in triplicates, and (**I**) cell lysates were analyzed for cleaved PARP and cleaved caspase-3 on immunoblots and normalized to β-actin levels. The representative immunoblots of experiments performed in duplicate are shown. PFU: plaque forming units; MOI: multiplicity of infection; RL RNAs: RNase L-cleaved small RNAs; Ctrl RNAs: control small RNAs; KD: knockdown * *p* < 0.05; ** *p* < 0.01; *** *p* < 0.001; ns: not significant.

CVB3 infection of cells activates autophagy to promote replication and, in later stages, induces cell death to release virions. We and others showed that the activation of RNase L induces autophagy, and the RNase L-cleaved RNAs promote the switch to apoptosis [59,63,76]. However, the role of RNase L in the CVB3 life cycle is not known. HeLa-M cells are deficient in endogenous RNase L and allow us to study the unique contribution of RL RNAs in CVB3 infection. HeLa-M cells stably expressing RNase L and control cells lacking RNase L were infected with CVB3 and viral titer in the supernatant and copies of CVB3 genomic RNA in the supernatant were determined. Cells expressing RNase L restricted CVB3 replication, as the viral titer was reduced by 30% compared to parental cells lacking RNase L (Figure 9A). Higher viral RNA copy numbers were obtained in cells lacking RNase L compared to cells stably expressing RNase L (Figure 9B). The effect of RNase L on CVB3 correlated with increased IFN-β produced by HeLa-M RNase L cells, suggesting an antiviral effect of RNase L on CVB3 infection (Figure 9C). To determine how cell death induced by RL RNAs impacts CVB3 infection, HeLa-M cells transfected with RL RNAs or control RNAs were infected with CVB3, the RNA copy numbers were determined from the culture supernatant, and cell lysates were harvested and analyzed for the cleavage of apoptotic markers, PARP, and caspase-3. Compared to control RNA and untreated cells, the viral RNA copies were significantly reduced in cells treated with RL RNAs (Figure 9D). Cells infected with CVB3 showed the cleavage of PARP and caspase-3, and the levels were increased in cells treated with RL RNAs (Figure 9E). These data suggest that apoptosis induced by RL RNAs contributed to reduced CVB3 replication.

We tested the role of apoptosis by RL RNAs in CVB3 infection in two settings: (a) inhibiting caspase-3 activity using a specific caspase-3/7 inhibitor (Ac-DEVD-CHO) and (b) using IRF-3 KD cells wherein the transcriptional induction of IFN and transcription-independent cell death are inhibited. Inhibiting caspase-3 activity resulted in a dramatic increase in CVB3 RNA copies, and cells treated with RL RNA in the presence of the inhibitor showed a smaller increase compared to control RNA-treated cells. The viral titer determined by RNA copies was comparable in mock-treated and control RNA-treated cells (Figure 9F). CVB3-infected cells showed an increase in the cleavage of PARP and caspase-3, which was further increased by RL RNAs, and this effect was abolished in the presence of the caspase-3/7 inhibitor (Figure 9G). The smaller increase in response to RL RNAs in the presence of caspase-3 inhibitors suggest that alternative non-apoptotic cell death may also affect CVB3 replication [56]. To rule out the effect of RL RNA-induced IFN signaling mediated by Rig-I and induced by transcription factor IRF-3, we knocked down IRF-3 in HeLa cells treated with RL RNAs or control RNAs followed by CVB3 infection and determined the impact on CVB3 pathogenesis. Knocking down IRF-3 also eliminates the IRF-3-Bax-induced apoptosis in these cells. The lack of IRF-3, as expected, facilitated robust CVB3 replication, and in these cells, RL RNAs was able to significantly inhibit CVB3 replication, indicating an IRF-3-independent effect (Figure 9H). On immunoblots, IRF-3KD cells treated with RL RNAs and CVB3 showed an increase in caspase-3 cleavage compared to IRF-3 expressing cells (Figure 9I), further supporting our observations on the dispensable role of apoptosis by IRF-3. These results suggest that inducing premature cell death by RL RNAs affects CVB3 replication, resulting in a decrease in viral titers, and inhibiting apoptosis by caspase-3 inhibitors facilitates the robust completion of CVB3 infection and the release of virions.

## 4. Discussion

RNase L is activated by a unique ligand, 2-5A, that is produced in virus-infected cells and exerts antiviral effects by targeting diverse RNA substrates. The cleavage of ssRNAs of viral and cellular origin, including mRNAs and rRNAs, directly eliminate viral genomes and replication intermediates or affect translation machinery that viruses require to complete the viral life cycle [50]. Recent studies have shown that RNase L activity induces a ribotoxic stress response, possibly through ribosome collisions, involving ZAK α and stress kinases, resulting in cell death as a viral clearance mechanism [56,57]. In addition, the nucleolytic activity of RNase L results in cleavage products, RL RNAs, that are short dsRNAs with 5′-hydroxyl and 2′-3′ cyclic phosphate termini and participate in signaling events with broad antiviral effects by promoting stress granule formation to amplify IFN production, activate inflammasome in immune cells, induce autophagy, and promote switch to apoptosis by targeting Beclin-1 cleavage [35,59,61]. These effects of RL RNAs are mediated by dsRNA receptors that vary in expression levels and in a cell-type-specific manner, resulting in a wide spectrum of antiviral responses. For instance, RL RNAs bind to RLR helicases Rig-I and MDA5 to amplify IFN production, and they bind the RNA helicase DHX33 in immune cells to activate inflammasomes involving MAVS but independent of Rig-I. In this study, we identify the requirement of the Rig-I and RNA helicase DHX15 in inducing apoptosis by RL RNAs. Both proteins are required for inducing apoptosis, and the signals are transduced by the mitochondrial adaptor MAVS and stress kinases JNK and P38. Transcription factors IRF-3 and NF-kB, while required for producing IFN and proinflammatory cytokines by RL RNAs, are dispensable for inducing apoptosis. Using RNA binding defective mutants, we show that RNA binding by both DHX15 and Rig-I is required to induce apoptosis. Inducing premature cell death by RL RNAs exacerbates the RNase L antiviral effect in CVB3 replication. Taken together, our results suggest that the activation of RNase L in virus-infected cells promotes antiviral effects by signaling events engaging different dsRNA receptors in overlapping and intersecting pathways with distinct outcomes to produce cytokines or modulating cell death.

RNase L activity can impact apoptosis induction at multiple levels. Recent studies showed that the cleavage of mRNA in actively translating polysomes may result in ribosome collisions, activating a ZAK α and ribotoxic stress response involving JNK and P38 kinases, culminating in apoptosis [56]. Our previous studies showed the induction of autophagy as a prosurvival mechanism in response to direct RNase L activation by 2-5A [63,76]. Inhibiting autophagy and the cleavage products of RNase L resulted in apoptosis by the caspase-3-mediated cleavage of beclin-1 [59]. The requirement of JNK is a common feature in all instances and has also been reported in apoptosis by RNase L in response to chemotherapy drugs [60,69]. While the signaling pathways underlying apoptosis were well characterized, the dsRNA sensors that sense the RL RNAs to promote apoptosis were not defined. Using cell lines genetically deleted for dsRNA PRRs, like PKR, Rig-I, OAS1, OAS2, and OAS3, and the knockdown cells of DHX15 and MDA5, we showed that both Rig-I and DHX15 are required for apoptosis by RL RNAs. We showed previously that RL RNAs activate PKR to form antiviral stress granules to mount an IFN response; that PKR is dispensable for apoptosis was unexpected, as its role in apoptosis by synthetic dsRNA polyI:C has been widely reported [62,77,78]. In tumor cells, Rig-I ligands were shown to induce apoptosis involving the OAS/RNase L pathway [25], while cells ablated for OAS isoforms did not rescue cell death in response to RL RNAs. Our observations suggest that, at the biochemical level, immune signaling and apoptosis induction by RL RNAs are distinct from other dsRNA effectors.

Previous studies have identified DHX15 as a co-receptor in RLR signaling, wherein DHX15 engages in PAMP binding but does not participate in signaling events independently, as it lacks the CARD domain; instead, it interacts with RIG-I to enhance RLR signaling in response to RNA virus infection [42]. In our studies, DHX15 interacts with Rig-I and MAVS in response to RNase L activation by 2-5A and in cells transfected with RL RNAs, and this complex assembly can mediate RLR signaling. In the context of apoptosis, cells lacking either DHX15, Rig-I, or both show increased survival in response to RL RNAs, and the overexpression of DHX15 does not induce apoptosis in MAVS KO cells, indicating that MAVS is essential for apoptosis and functions downstream of the Rig-I/DHX15 signaling, in contrast with earlier publication that suggests DHX15 functions downstream of MAVS (Figure 3) [39]. Using a panel of DHX15 mutants generated based on crystal structure, we identified amino acid residues that were critical for dsRNA binding. The expression of the RNA binding mutants of Rig-I(K858/861A) in Rig-I KO cells or DHX15(R194/195A) in DHX15 KD cells promoted cell survival in response to RL RNAs, indicating the requirement of RNA binding to effect apoptosis. More importantly, the presence of endogenous Rig-I or DHX15 in single gene ablated cells was unable to promote cell death, indicating the need of both proteins, possibly enabling a complex formation that mediates cell death. This conclusion is further supported by the expression of both RNA binding mutants of Rig-I and DHX15 in cells lacking both proteins (dKO). The cleavage of PARP and caspase-3 was significantly reduced compared to cells expressing WT proteins (Figure 8J), and cells were more viable. These observations suggest that the two receptors may share similar ligand requirements, and binding by both proteins is required for cell death. However, a small amount of cell death remained in DKO cells, possibly due to the residual activity of DHX15 in knockdown DKO cells or other alternate cell death mechanisms. Our data suggest that RNase L activation and the cleaved RNAs facilitate Rig-I/DHX15 interaction, which, in turn, recruits MAVS to activate apoptosis.

The activation of JNK and P38 has been shown in many cell types during RNase L activation, and recent studies show that ZAK α kinase activity is required for JNK and P38 activation [56]. In RL-treated cells, we show that JNK and P38 phosphorylation is reduced significantly in Rig-I KO/DHX15 KD cells and cells where JNK and P38 activity is inhibited, which correlates with increased cell survival. In *Drosophila* cells, DHX15 was identified as an activator of JNK, P38, and NF-kB pathways in response to polyI:C and infection with an RNA virus to mediate cytokine production and apoptosis [39]. In myeloid dendritic cells, DHX15 was identified as a dsRNA binding protein to activate an immune response to RNA viruses by interaction with MAVS [40]. In line with these observations with polyI:C, our data suggest that RL RNAs activate JNK and P38 to induce apoptosis in a Rig-I- and DHX15-dependent manner.

RLR signaling activates IRF-3 and NF-kB transcription factors downstream of MAVS to produce type I IFN and proinflammatory cytokines. The cleavage products of RNase L bind Rig-I/MDA5 and signal through MAVS, IRF-3, and NF-kB to amplify IFN production [61]. The activation of IRF-3 by dsRNA signaling induces apoptosis by the intrinsic mitochondrial pathway and the cleavage of caspase-3 and PARP, which is independent of transcriptional activity [29]. Interestingly, RL RNA-induced cell death is IRF-3-independent, as cells lacking IRF-3 showed no significant difference in apoptosis compared to WT cells. NF-kB is activated in HT1080 cells by 2-5A and RL RNAs as evidenced by the nuclear translocation of p65 subunit and expression of proinflammatory cytokines [62]. Inhibiting NF-kB activity by expressing the IkB super-repressor did not impact apoptosis by RL RNAs, indicating that NF-kB activity, while required for expression of target genes and proinflammatory cytokines, was not required for RNase L-induced apoptosis. Accordingly, the induction of IFNβ and proinflammatory cytokines by RL RNAs was significantly reduced in cells lacking either Rig-I or DHX15 or both proteins. These results demonstrate that IFN and cytokine production and the induction of apoptosis by RL RNAs are distinct events downstream of the recognition of RL RNAs by Rig-I and DHX15. Future investigations will address whether a subset of RL RNAs with unique features specify cytokine induction vs. apoptosis and if the effects are dictated in a cell-type-specific manner, depending on the abundance and localization of the RNA binding receptors. In that regard, we showed that RL RNAs induce apoptosis in primary human fibroblasts (Figure S3) and that the effects are not limited to tumor cell lines.

The elimination of virus-infected cells by apoptosis is a conserved antiviral mechanism, and several viruses, including CVB3, co-opt and use apoptosis to facilitate dissemination. We examined the impact of RL RNA-induced cell death on CVB3 production. We first demonstrated that CVB3 is susceptible to the RNase L antiviral effect, and the effect correlates with increased IFNβ production. Inducing premature apoptosis by transfecting RL RNAs in cells lacking RNase L expression significantly dampened CVB3 replication. Importantly, CVB3 titer was increased by the inhibition of apoptosis by caspase-3 inhibitor treatment. Our results suggest that the RL RNA-activated antiviral effect by inducing apoptosis early before the viral life cycle is completed and inhibiting apoptosis allows the virus to use the host cells for continued proliferation to promote a more robust infection. CVB3 infection of IRF-3 KD Hela cells allowed CVB3 replication to very high titers, as would be expected for cells lacking the antiviral effect, and RL RNA induced cell death that reduced CVB3 titers, even in cells lacking IRF-3. These results further support our observations that IRF-3, while necessary for IFN production, does not mediate RL RNA-induced apoptosis.

In summary, our observations suggest a broad impact of RNase L in virus-infected cells. RNase L activation by a unique ligand 2-5A produced only in virus-infected cells results in the direct degradation of viral and cellular RNAs activating a ribotoxic stress response pathway as an early response. The simultaneous induction of autophagy is likely a byproduct of stress imposed by infection, allowing infected cells to maintain cellular homeostasis. In addition, RNase L nucleolytic activity produces cleaved RNAs with immunostimulatory properties that can activate immune response, engaging diverse cell-type-specific receptors and biochemical features of the cleaved products. The heterogeneity in the viral and cellular cleaved products combined with the availability of PRRs results in the activation of multiple overlapping signaling pathways, expanding the scope and timeline of the antiviral effect. In our study, we show that the induction of cytokines and apoptosis induction are independent events activated by RL RNAs downstream of the binding RNA helicases.

## Figures and Tables

**Table 1:** sgRNA sequence for OAS1, OAS2, and OAS3 knockout using CRISPR/Cas9 system.

Primer	Sequence
sgOAS1-F	5′ CACCGCAGGATCAGTTAAATCGCCG 3′
sgOAS1-R	5′ AAACCGGCGATTTAACTGATCCTGC 3′
sgOAS2-F	5′ CACCGGAAGCTGGGTTGGTTTATCC 3′
sgOAS2-R	5′ AAACGGGATAAACCAACCCAGCTTCC 3′
sgOAS3-F	5′ CACCGGCGATGCCCGCATCTCACTG 3′
sgOAS3-R	5′ AAACCAGTGAGATGCGGGCATCGCC 3′

**Table 2:** Primers for site-directed mutagenesis.

Mutation	Orientation	Primer Sequence
R22A	Sense	5′-cactactgcagtcttcaaatgcaatggagtaaccaacttcct-3′
	Antisense	5′-aggaagttggttactccattgcatttgaagactgcagtagtg-3′
R243A	Sense	5′-gggatcattcatagcttcagcaagtaacatcccatcagtcatat-3′
	Antisense	5′-atatgactgatgggatgttacttgctgaagctatgaatgatccc-3′
T237A	Sense	5′-ttcacgaagtaacatcccatcagccatatacttaagaatggtttttg-3′
	Antisense	5′-caaaaaccattcttaagtatatggctgatgggatgttacttcgtgaa-3′
RR194/195AA	Sense	5′-acactcattgcagccactgccgcgggttgggtacaggcaac-3′
	Antisense	5′-gttgcctgtacccaacccgcggcagtggctgcaatgagtgt-3′
T421/N422AA	Sense	5′-gtcaaagacgtctctgctatggcagctgacacaactacctttcttcc-3′
	Antisense	5′-ggaagaaaggtagttgtgtcagctgccatagcagagacgtctttgac-3′

**Table 3:** Primer sequences for real-time RT-PCR.

Primer	Sequence (5′-3′)
IFN-β F	GGAGGACGCCGCATTGAC
IFN-β R	TGATAGACATTAGCCAGGAGGTTC
CCL5 F	CCAGCAGTCGTCTTTGTCAC
CCL5 R	CTCTGGGTTGGCACACACTT
IL-8 F	AAGAGAGCTCTGTCTGGACC
IL-8 R	GATATTCTCTTGGCCCTTGG
IP-10 F	TTCCTGCAAGCCAATTTTGTC
IP-10 R	TCTTCTCACCCTTCTTTTTCATTGT
GAPDH F	GCAAATTCCATGGCACCGT
GAPDH R	TCGCCCCACTTGATTTTGG
CVB3 F	CACACTCCGATCAACAGTCA
CVB3 R	GAACGCTTTCTCCTTCAACC

## Data Availability

The original contributions presented in the study are included in the article, further inquiries can be directed to the corresponding author.

## Supplementary Materials

The following supporting information can be downloaded at: https://www.mdpi.com/article/10.3390/v16121913/s1, Figure S1: Expression of proteins in knockout and knockdown cells. (A) CRISPR/Cas9 knockout of *OAS1*, *OAS2* and *OAS3* in HT1080 cells, (B) Knockout or knockdown of Rig-I and DHX15 in HT1080 cells. Figure S2: Analysis of DHX15KD cells. (A) Knockdown efficiency of DHX15 in knockdown cells, (B) Cell viability in the DHX15KD cells. Figure S3: RNase L-cleaved RNAs induce apoptosis in primary NuFF cells.
