# Supplementary material for: DHX15 and Rig-I Coordinate Apoptosis and Innate Immune Signaling by Antiviral RNase L
Source: Viruses. 2024 Dec 13;16(12):1913. doi: 10.3390/v16121913 (PMC11680366; doi:10.3390/v16121913)
Supplement: Supplementary file 1 [file viruses-16-01913-s001.zip › viruses-3310933-supplementary.pdf]

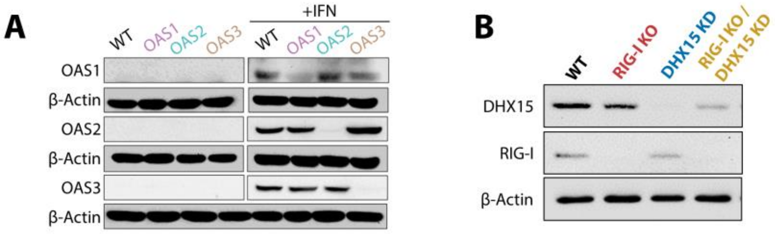

Supplementary Figure S1: (A) CRISPR/Cas9 knock out of OAS1, OAS2 or OAS3 and HT1080 WT cells were mock treated or treated with IFNβ (1000u/ml) for 16h. Cell lysates were analyzed on immunob-lots with antibodies against OAS1, OAS2 or OAS3 and normalized to β-actin levels. (B) Expression levels of DHX15 and Rig-I was determined on immunoblots in HT1080 WT, Rig-I KO, DHX15 KD and Rig-I KO/DHX15 KD cell lysates and normalized to β-actin levels.

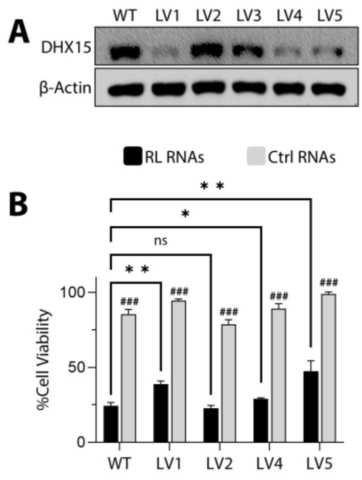

Supplementary Figure S2: HT1080 cells were transduced with DHX15 targeting shRNA lentivirus constructs and knockdown efficiency was determined on immunoblots with DHX15 antibodies and normalized to β-actin levels. DHX15 KD clones from the above analysis were transfected with RL RNAs or Ctrl RNAs (2μg/mL) and percent cell viability was determined by MTT assay. Induction by RL RNAs in the various LV knockdown cells were compared to WT and significance shown as *. Induction by RL RNAs compared to ctrl RNAs within a cell type is indicated by #. LV: Lentivirus; RL RNAs: RNase L-cleaved small RNAs; Ctrl RNAs: Control small RNAs; * $p < 0.05$; ** $p < 0.01$; ns: not significant, ###$p<0.001$.

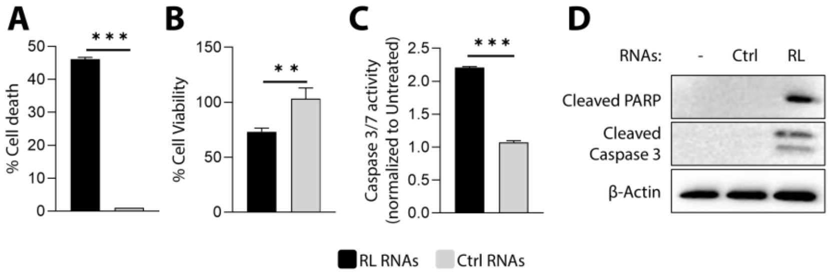

Supplementary Figure S3: RNase L-cleaved small RNA induces apoptosis in primary cells. Primary NuFF cells were transfected with RL RNAs or Ctrl RNAs (2μg/mL) and (A) percent cell death by trypan blue exclusion, (B) percent cell viability by MTT assay, and (C) Caspase-3/7 activity was de-termined. (D) Cell lysates were analyzed for cleaved PARP and Cleaved Caspase-3 on immunoblots and normalized to β-actin levels. Results shown represent mean ± SD for the experiment performed in triplicate. RL RNAs: RNase L-cleaved small RNAs; Ctrl RNAs: Control small RNAs; ** $p < 0.01$; *** $p < 0.001$.
